# Serotype-specific evolutionary patterns of antimicrobial-resistant *Salmonella enterica*

**DOI:** 10.1186/s12862-019-1457-5

**Published:** 2019-06-21

**Authors:** Jingqiu Liao, Renato Hohl Orsi, Laura M. Carroll, Jasna Kovac, Hongyu Ou, Hailong Zhang, Martin Wiedmann

**Affiliations:** 1 Department of Food Science, 341 Stocking Hall, Cornell University, Ithaca, NY 14853 USA; 2 Graduate Field of Microbiology, Cornell University, Ithaca, NY 14853 USA; 3 Department of Food Science, The Pennsylvania State University, University Park, PA 16802 USA; 4 School of Life Sciences & Biotechnology, Shanghai Jiao Tong University, Shanghai, 200240 China; 5 Department of Computer Science & Engineering, Ohio State University, Columbus, OH 43210 USA

**Keywords:** *Salmonella enterica*, Serotypes, Antimicrobial resistance, Genome decay, Positive selection, Homologous recombination

## Abstract

**Background:**

The emergence of antimicrobial-resistant (AMR) strains of the important human and animal pathogen *Salmonella enterica* poses a growing threat to public health. Here, we studied the genome-wide evolution of 90 *S. enterica* AMR isolates, representing one host adapted serotype (*S.* Dublin) and two broad host range serotypes (*S.* Newport and *S.* Typhimurium).

**Results:**

AMR *S.* Typhimurium had a large effective population size, a large and diverse genome, AMR profiles with high diversity, and frequent positive selection and homologous recombination. AMR *S.* Newport showed a relatively low level of diversity and a relatively clonal population structure. AMR *S.* Dublin showed evidence for a recent population bottleneck, and the genomes were characterized by a larger number of genes and gene ontology terms specifically absent from this serotype and a significantly higher number of pseudogenes as compared to other two serotypes. Approximately 50% of accessory genes, including specific AMR and putative prophage genes, were significantly over- or under-represented in a given serotype. Approximately 65% of the core genes showed phylogenetic clustering by serotype, including the AMR gene *aac (6′)-Iaa*. While cell surface proteins were shown to be the main target of positive selection, some proteins with possible functions in AMR and virulence also showed evidence for positive selection. Homologous recombination mainly acted on prophage-associated proteins.

**Conclusions:**

Our data indicates a strong association between genome content of *S. enterica* and serotype. Evolutionary patterns observed in *S*. Typhimurium are consistent with multiple emergence events of AMR strains and/or ecological success of this serotype in different hosts or habitats. Evolutionary patterns of *S*. Newport suggested that antimicrobial resistance emerged in one single lineage, Lineage IIC. A recent population bottleneck and genome decay observed in AMR *S.* Dublin are congruent with its narrow host range. Finally, our results suggest the potentially important role of positive selection in the evolution of antimicrobial resistance, host adaptation and serotype diversification in *S. enterica*.

**Electronic supplementary material:**

The online version of this article (10.1186/s12862-019-1457-5) contains supplementary material, which is available to authorized users.

## Background

*Salmonella enterica*, the causative agent of salmonellosis, is a human and animal pathogen that causes substantial economic losses and major public health concerns worldwide [1, 2]. Salmonellosis is estimated to be responsible for 93.8 million global human cases annually, among which 80.3 million cases are estimated to be foodborne [3]. In the United States alone, salmonellosis contributes to approximately 1.2 million human illnesses [4] and medical costs associated with salmonellosis total $3.7 billion every year [5]. The emergence and global spread of antimicrobial-resistant (AMR) *S. enterica* have further raised the public concern, as AMR *S. enterica* compromise the ability to treat infections in humans and animals [6, 7]. In addition, previous studies have suggested that AMR strains of *S. enterica* may be more virulent than susceptible ones [8].

*S. enterica* contains > 2500 recognized serotypes, which display a broad range of epidemiological and ecological characteristics. Host-adapted serotypes typically induce systemic disease in a limited number of host species, while non-host-adapted serotypes usually cause self-limiting gastroenteritis and less commonly systemic disease in a wide range of hosts [9]. Previous studies [10–12] have provided initial evidence that *S. enterica* serotype differences in host ranges and virulence characteristics are associated with genomic characteristics (e.g., genetic diversity, gene presence and absence patterns). These genomic characteristics are consequences of a variety of evolutionary and population genetics processes, such as gene acquisition and deletion, positive selection, homologous recombination and changes in population size. In particular, acquisition of non-homologous novel genes (e.g., pathogenicity islands, antibiotic resistance genes) by plasmid- or phage-mediated horizontal gene transfer, has been demonstrated to play a critical role in the evolution of *Salmonella* [8, 13]. Previous studies have also indicated that the level of gene degradation and gene deletion loosely correlates with the degree of host specificity displayed by particular *Salmonella* serotypes [14]. Loss of key metabolic functions has specifically been observed in many host-restricted serotypes, such as *S*. Typhi and *S*. Paratyphi A (human), and *S*. Gallinarum (fowl) [14–16]. Besides gene acquisition and deletion, positive selection and homologous recombination have been shown to play important roles in the serotype divergence and adaptive evolution in *Salmonella* as well [15, 17, 18]. For example, a total of 41 *Salmonella* genes reported by [15] showed evidence for positive selection, including genes likely contributing to virulence. In addition, *S.* Typhi and *S.* Paratyphi appear to have experienced a burst of recombination involving a quarter of their genes during the course of their adaptation to a highly virulent and human-specific lifestyle [19]. Changes in effective population size (*Ne*) can also have important impacts on emergence and diversification of bacterial lineages [20]. For example, multiple independent population expansions have been reported to lead to radial clusters of haplotypes among *S*. Typhi in Asia and Africa [21].

While a number of studies have explored the evolution of *Salmonella*, many of these studies have focused on a few specific serotypes (often *S.* Typhi) [16, 21], and few studies [6, 7, 16, 22] have specifically used genomics approaches to explore the evolutionary history and population genetic structure of AMR and multidrug-resistant (MDR) *Salmonella*, especially the potential influence of processes such as positive selection on the evolution of its antibiotic resistance*.* A few specific *Salmonella* serotypes are of a particular concern with regard to emergence and spread of MDR strains, including, but not limited to, serotypes Typhimurium, Newport, and Dublin, which represent the three serotypes this study focused on. As a pathogen infecting a wide range of hosts, *S.* Typhimurium is one of the leading causes of gastroenteritis in humans, and it is able to produce systemic infection in calves, sheep, goats, and pigs [9, 23, 24]. MDR *S.* Typhimurium phage type DT 104 represents one of the first MDR *Salmonella* clonal groups that was considered as a particular public health concern. This clonal group is characterized by a typical pattern of penta-resistance to ampicillin, chloramphenicol, streptomycin, sulfonamide, and tetracycline, and appears to have emerged in the United Kingdom as early as 1980 with global dispersal since the 1990s [25, 26]. In addition, another MDR Typhimurium clonal group mainly represented by phage type DT 193 has been described [27]. Most DT 193 isolates show the same pattern of penta-resistance as DT 104 except for kanamycin resistance in DT 193 (instead of chloramphenicol resistance in DT 104) [27]. *S.* Newport is a non-host-adapted pathogen that has been linked to many human gastroenteritis cases in both the United States and Europe in the past decades [18, 28]. *S.* Newport has been shown to be polyphyletic and *S.* Newport populations have been reported to have a geographic structure [2]. In a previous study, *S.* Newport strains isolated from Europe were more likely to belong to Lineage I, while strains from North America were more likely to belong to Lineages II and III [18]. A widespread emergence of Newport-MDRAmpC strains has been documented in the United States, which largely contributed to a 5-fold increase in the prevalence of *Salmonella* resistant to expanded-spectrum cephalosporins between 1998 and 2001 [29]. As a host-adapted serotype, *S.* Dublin is highly adapted to cattle and rarely infects humans [14]. In Japan, the prevalence of *S.* Dublin has drastically increased after the acquisition of a resistance (R)-plasmid in the early 1980s; this plasmid confers resistance to multiple antibiotics, including ampicillin, kanamycin, and nalidixic acid. [30]. In the United States, the incidence rate for human infection with *S.* Dublin increased more than that for infection with other serotypes, and a higher proportion of isolates were resistant to > 7 classes of antibiotics during 2005–2013 than during 1996–2004 [31]. Consistent with the host specificity of *S.* Dublin, previous studies suggested that at least some antimicrobial resistance traits in *S.* Dublin, such as resistance to nalidixic acid, were acquired within the bovine reservoirs [30, 32]. While our study reported here focused on characterization of AMR *S.* Typhimurium, *S.* Newport, and *S.* Dublin strains, emergence of AMR and MDR has also been described in other serotypes including Heidelberg and Paratyphi B [33].

In order to better understand the serotype-specific microevolution patterns of AMR *S. enterica*, we employed comparative genomic, evolutionary and phylogenetic approaches to further analyze previously reported [7] genome sequences for 90 AMR *S.* Dublin, *S.* Newport, and *S.* Typhimurium isolates from dairy cattle and humans from Washington state and New York state. The specific aims of this study were i) to characterize the evolutionary history of AMR *S.* Dublin, *S.* Newport, and *S.* Typhimurium; ii) to assess the relationship between genome content, including AMR genes and putative prophage genes, and serotype; iii) to identify genomic characteristics associated with the narrower host range of *S.* Dublin compared to other two serotypes; iv) to explore the influence of population dynamics, homologous recombination, and positive selection on the evolution of AMR *S. enterica.*

## Results

### Pan and core genomes, and AMR and prophage gene diversity

**Fig. 1:**
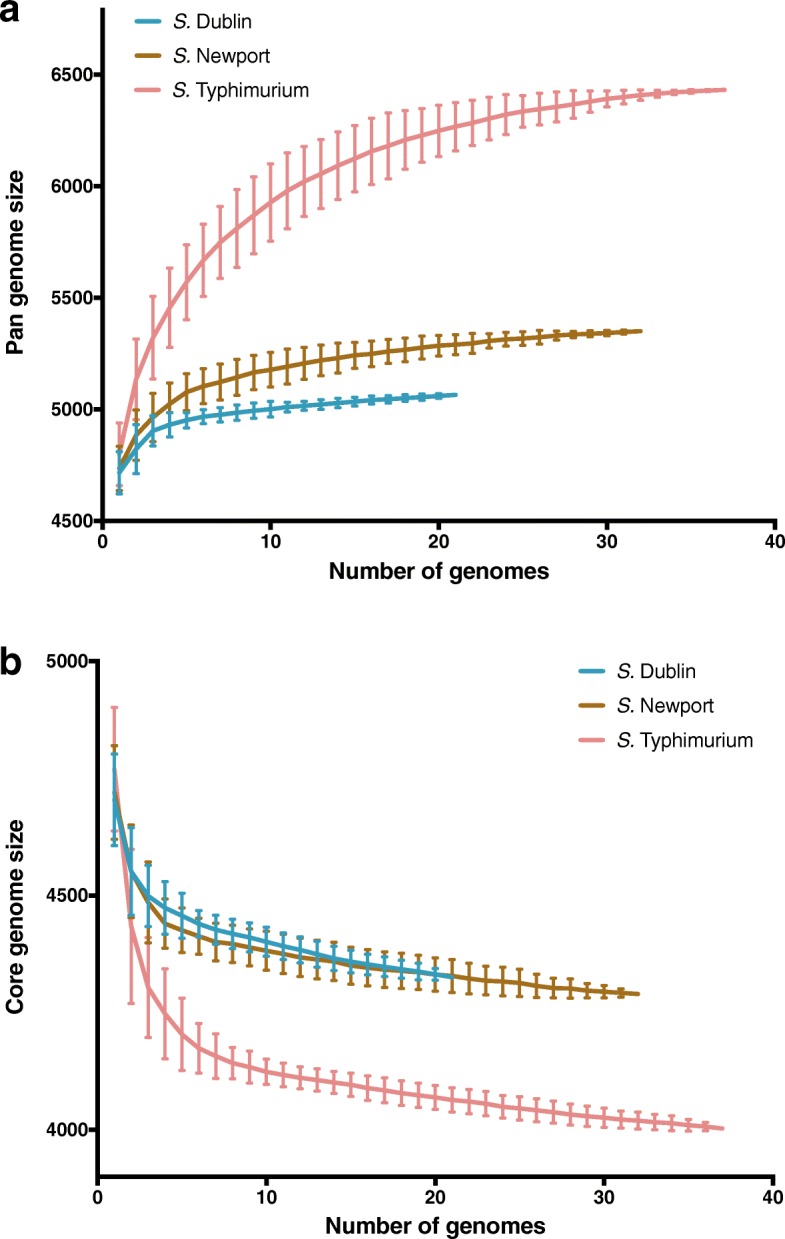
Accumulation curves of **a** pan genome and **b** core genome of AMR *S.* Dublin (in blue), *S.* Newport (in brown) and *S.* Typhimurium (in red). The vertical bars indicate the standard deviations based on 100 repetitions with randomization of the order of the genomes

**Fig. 2:**
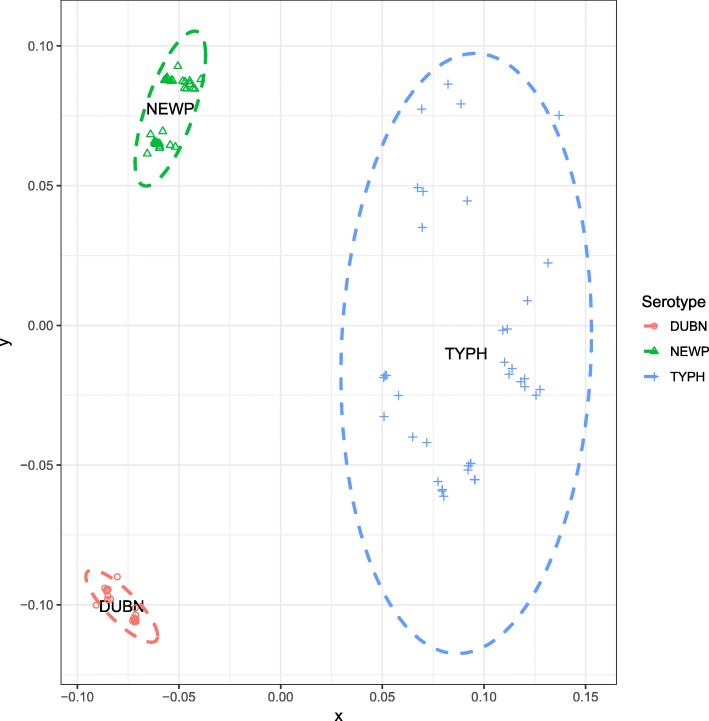
Non-metric multidimensional scaling ordination of AMR *S.* Dublin (in red), *S.* Newport (in green) and *S.* Typhimurium (in blue) isolates based on gene presence/absence

While the 90 genomes analyzed here were previously reported and used to assess associations between isolate sources and selected phenotypic (e.g., AMR) and genetic characteristics (e.g., presence/absence of AMR genes and plasmid replicons) [7], these genomes had not been previously annotated. In order to facilitate in-depth evolutionary and population genetics analyses of these genomes, we initially annotated these 90 genomes. Annotation identified a total of 7077 orthologous genes across the three serotypes (Additional file 1: Table S1). The pan genome of AMR *S.* Typhimurium was larger (by > 1000 orthologous genes) than that of *S.* Dublin and *S.* Newport, and the core genome of AMR *S.* Typhimurium was smaller (by approximately 300 orthologous genes) than that of the other two serotypes (Additional file 1: Table S2). According to the accumulation curves of the pan and core genome (Fig. 1a and b), *S.* Typhimurium displayed steeper slopes with an increasing number of sampled genomes affecting the number of genes in the pan and core genomes more drastically in this serotype, as compared to *S.* Dublin and *S.* Newport. Non-metric multidimensional scaling clustered isolates by serotype and showed a looser clustering of *S.* Typhimurium isolates, indicating that the three serotypes have very different gene presence/absence patterns and that *S.* Typhimurium has a more diverse gene composition (Fig. 2). The genome size of AMR *S.* Dublin ranged from 4.92 to 5.02 Mb; that of *S.* Newport ranged from 4.84 to 5.03 Mb; and that of *S.* Typhimurium ranged from 4.87 to 5.25 Mb (Additional file 1: Table S3).

Consistent with previous genome analysis [7], 41 different AMR genes were identified among the three serotypes (Additional file 1: Table S4). While genes encoding penicillin-binding proteins (PBPs) were included as AMR genes in [7], they were excluded here. PBP is the target of β-Lactams and although mutations in PBP genes have been shown to confer resistance to β-Lactams in *S. enterica* [34], the presence of PBP genes is not associated with resistance to β-Lactams. *S.* Typhimurium isolates exhibited a large range in the number of AMR genes with 3 to 22 annotated AMR genes in a given isolate (Additional file 2: Figure S1) and a higher overall diversity of AMR genes (Shannon-wiener index: 3.01) as compared to *S.* Dublin and *S.* Newport (Shannon-wiener index of 1.73 and 1.05, respectively). A total of 732 prophage protein groups (PPGs) were identified, including 44 PPGs present in all 90 genomes (Additional file 1: Table S5). AMR *S.* Typhimurium genomes showed a wider range in the number of annotated PPG found in a given isolate, as compared to *S.* Dublin and *S.* Newport (Additional file 2; Figure S2).

### Population dynamics

**Fig. 3:**
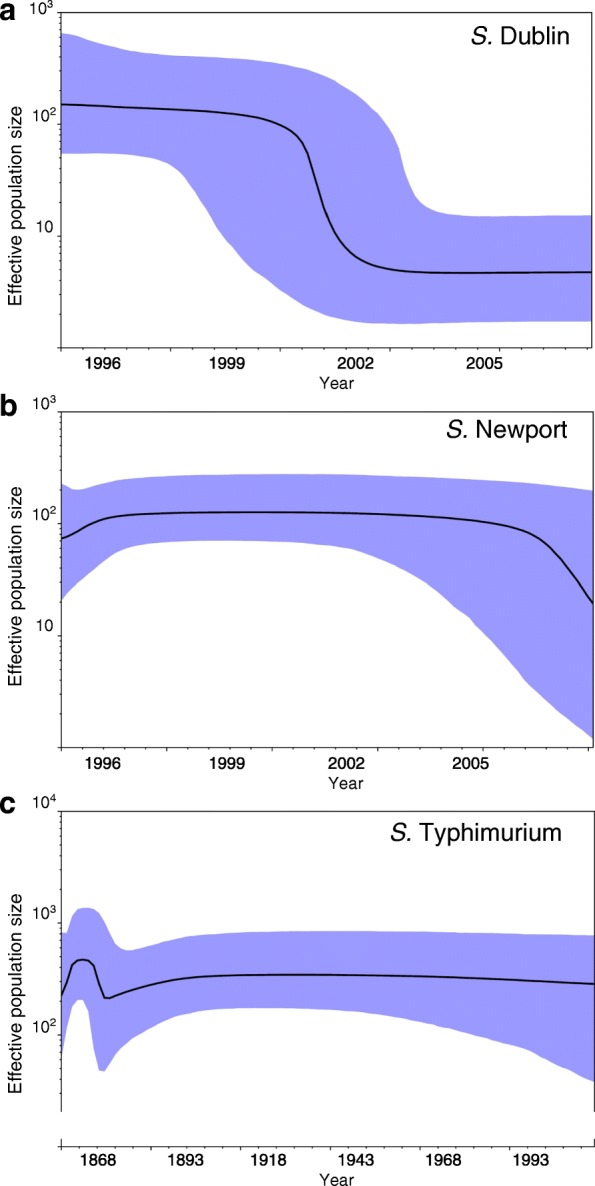
Bayesian skyline plot indicating changes in the effective population size (*Ne*) of **a** AMR *S.* Dublin, **b** *S.* Newport and **c** *S.* Typhimurium over time with a relaxed molecular clock. The shaded area represents the 95% confidence intervals

In order to estimate population changes over time, four Markov chain Monte Carlo (MCMC) population models, built based on single nucleotide polymorphism (SNP) data, were compared. Core SNPs were identified within each of the three serotypes including 2725 in *S.* Typhimurium, 128 in *S.* Dublin and 253 in *S.* Newport (numbers differed by 2 to 10 core SNPs from those previously reported by [7] as a different SNP filtering tool was applied). Among the four models evaluated, the Bayesian skyline population model, which estimates a posterior distribution of the *Ne* through time under a specified nucleotide-substitution model, showed the best fit for the population scenarios of AMR *S.* Dublin, *S.* Newport and *S.* Typhimurium. Bayesian skyline plots, which show the changes in the *Ne* over time, suggest a recent *Ne* reduction for *S.* Dublin (Fig. 3a). The *Ne* of *S.* Newport remained relatively constant over time, until very recently when it showed a noticeable decrease (Fig. 3b). The *Ne* of ancestral *S.* Typhimurium exhibited some fluctuations, followed by gradual stabilization with a constant *Ne* (Fig. 3c). Overall, the current AMR *S.* Typhimurium *Ne* is estimated to be approximately 10- and 50-fold greater than that for *S.* Newport and *S*. Dublin, respectively (Fig. 3).

### Serotype-associated accessory genes and GO terms, and pseudogene distribution

**Fig. 4:**
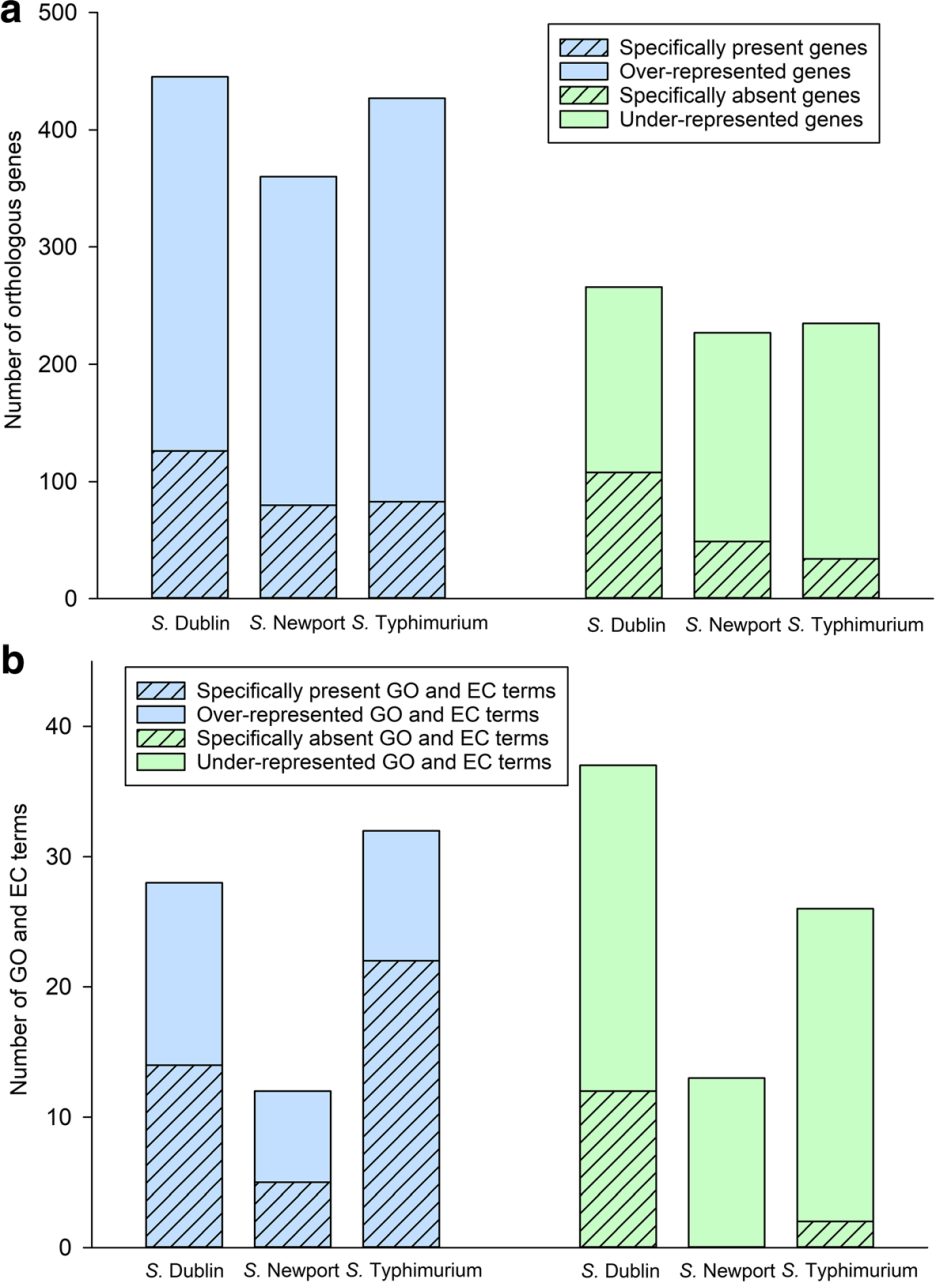
The over- and under- representation of **a** orthologous genes and **b** GO and EC terms in AMR *S.* Dublin, *S.* Newport, and *S.* Typhimurium. Orthologous genes and GO/EC terms over- / under- represented in one serotype are defined as the ones identified as significant in given comparisons to the other two serotypes (FDR < 0.05, odds ratio >6.71 or < 0.15, respectively, for over- / under- represented)

Among the 3440 accessory genes, 1725 were found to be serotype-associated (i.e., significantly over- or under- represented in a given serotype). A total of 445, 360, and 427 genes were significantly over-represented in AMR *S.* Dublin, *S.* Newport and *S.* Typhimurium isolates, including 126, 80, and 83 genes present in all AMR *S.* Dublin, *S.* Newport and *S.* Typhimurium, respectively (designated here as “specifically present” genes; Fig. 4a). A total of 266, 227 and 235 genes were significantly under-represented in AMR *S.* Dublin, *S.* Newport and *S.* Typhimurium, including 108, 49, and 34 genes absent in all AMR *S.* Dublin, *S.* Newport and *S.* Typhimurium, respectively (designated here as “specifically absent” genes; Fig. 4a). AMR *S.* Dublin had both the largest number of genes specifically present (including the gene encoding GntR family transcriptional regulator and multiple genes encoding type VI secretion proteins) as well as the largest number of genes specifically absent (including genes encoding some cytoplasmic proteins and transporters) (see Additional file 1: Table S6).

More specifically, distribution of AMR genes and putative prophage genes also differed among AMR *S.* Dublin, *S.* Newport and *S.* Typhimurium isolates. *aadB* and *cmlA* were significantly enriched in AMR *S.* Dublin, while *blaCARB*, *sulI*, *aadA*, *tetRG*, and *tetG* were significantly enriched in AMR *S.* Typhimurium (Table 1). *blaTEM-1D* was significantly under-represent in AMR *S.* Newport, while *blaCMY*, *sulII*, *tetA*, *tetR*, *strA*, and *strB* were significantly under-represented in *S.* Typhimurium (Table 1). Significant association of putative prophage genes (based on clustering of genes into PPGs) with serotypes (see Additional file 1: Table S9) seems to represent the presence of specific prophages in different serotypes. Specifically, AMR *S.* Dublin was dominated by PPG genes annotated as belonging to prophages Salmon_ST64B, Entero_Fels_2 and Entero_P22; AMR *S.* Newport was dominated by Gifsy_1, Salmon_SEN34 and Entero_PsP3, while AMR *S.* Typhimurium was dominated by Entero_ST104 and Salmon_ST64B (Additional file 1: Table S9).

**Table 1:** AMR genes significantly over- and under-represented in AMR *S.* Dublin, *S.* Newport, and *S.* Typhimurium

	Over-represented AMR genes^a^	Under-represented AMR genes^a^
*S.* Dublin	*aadB* *cmlA*	*–*
*S.* Newport	*–*	*blaTEM-1D*
*S.* Typhimurium	*blaCARB* *sulI* *aadA* *tetRG* *tetG*	*strB* *sulII* *strA* *tetR* *tetA* *CMY*

^a^AMR genes over- / under- represented in one serotype were defined as the ones identified as significant in given comparisons to the other two serotypes (FDR < 0.05, odds ratio >6.71 or < 0.15, respectively, for over- / under- represented)

At the gene ontology level, 4229 gene ontology (GO) terms (1878, 2020, and 331 classified into the molecular function, biological process, and cellular component categories, respectively) and 945 enzyme commission (EC) numbers were assigned to genes present in the 90 genomes (listed in Additional file 1: Table S7). While AMR *S.* Newport had the lowest number of both under- and over-represented GO terms and EC numbers, *S.* Typhimurium had the highest number of over-represented GO and EC terms and *S.* Dublin had the highest number of under-represented GO and EC terms (Fig. 4b, Additional file 1: Table S8). More specifically, a total of 14, 7, and 22 GO terms or EC numbers were only found (“specifically present”) in AMR *S.* Dublin, *S.* Newport, and *S.* Typhimurium, respectively, while 12, 0, and 2 GO terms or EC numbers were absent (“specifically absent”) in *S.* Dublin, *S.* Newport, and *S.* Typhimurium, respectively. Specifically present GO terms of particular interest included Type I site-specific deoxyribonuclease activity (GO:0009035), protein N-linked glycosylation via asparagine (GO:0018279), and antimonite transport (GO:0015699) in *S.* Newport, and inositol catabolic process (GO:0019310), response to hydrostatic pressure (GO:0051599), sphingolipid metabolic process (GO:0006665), and regulation of autophagy (GO:0010506) in *S.* Typhimurium. Specifically absent GO terms of particular interest included arginine transport (GO:0043858, GO:1902023) and ribulokinase activity (GO:0008741, EC:2.7.1.16) in *S.* Dublin.

Interestingly, AMR *S.* Dublin not only showed the highest number of specifically absent genes and GO terms, but also showed a significantly higher number of pseudogenes (average of 191 pseudogenes) as compared to AMR *S.* Newport and *S.* Typhimurium genomes (averages of 114 and 123 pseudogenes, respectively) (Additional file 2: Figure S3).

### Phylogeny and serotype-clustered core genes

**Fig. 5:**
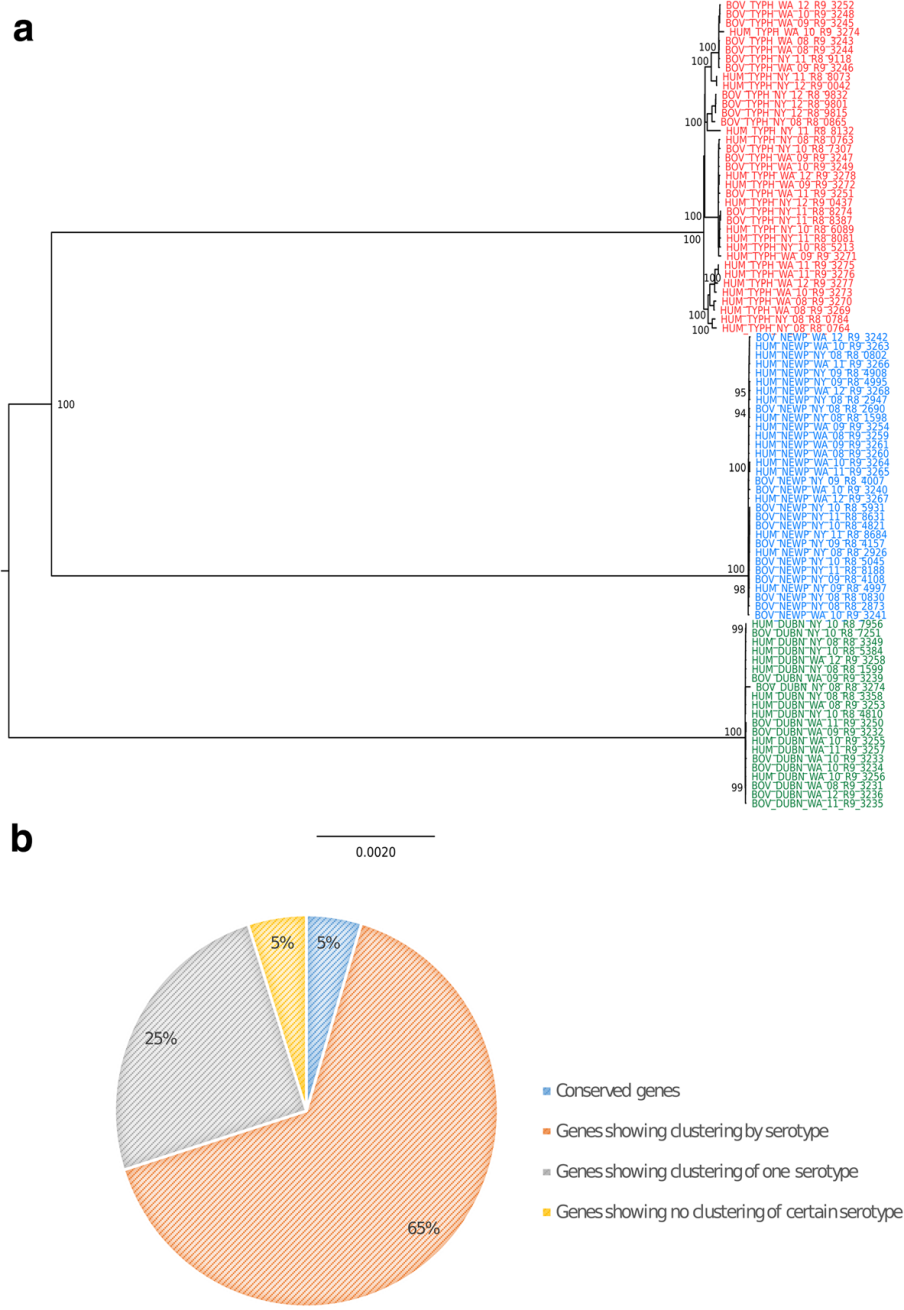
**a** Phylogenetic tree inferred by maximum likelihood method using the core genome SNPs of 90 *S. enterica* isolates. Tree is rooted by midpoint. Bootstrap values > 70% are presented on the tree. *S.* Dublin is indicated by green, *S.* Newport by blue, and *S.* Typhimurium by red. **b** Proportion of core genes with different phylogenetic clustering patterns. Genes showing a phylogenetic clustering by serotype on the tree are indicated in orange. Genes showing phylogenetic clustering of only one serotype (i.e., isolates of one serotype were grouping together while those of other two were not) are indicated in grey. Genes showing no particular clustering pattern are indicated in yellow. Conserved genes (i.e., all sequences were identical) are indicated in blue

Consistent with the previous study [7] on the 90 genomes analyzed here, a bootstrapped maximum likelihood (ML) tree, based on the 36,075 core SNPs identified across the 90 AMR *Salmonella* isolates, showed three well-supported major clades consistent with serotype assignment (Fig. 5a). A phylogeny constructed for the AMR *S*. Newport genomes included here, along with reference *S*. Newport genomes representing isolates of Lineage II (including sub-lineage A, B, C) and Lineage III [2], shows that all AMR *S.* Newport isolates included in this study belong to Lineage IIC (Additional file 2: Figure S5). Furthermore, comparison of the multilocus sequence typing (MLST) sequence types (STs) for the 37 AMR *S.* Typhimurium isolates studied here and DT 104 and DT 193 reference isolates showed that 30 of the 37 *S.* Typhimurium isolates have the same ST as the DT 104 reference isolates (ST-19), while 2 isolates have the same ST as the DT 193 reference isolate (ST-34) (Additional file 1: Table S10).

Individual ML trees for 2381 of the 3637 core orthologous genes also showed a phylogenetic clustering by serotype (these genes were designated as “serotype-clustered core genes”), indicating that a large fraction of core genes (65%) are serotype-clustered (Fig. 5b). Another 908 core genes exhibited a tree topology where isolates of one serotype clustered together, while isolates of the other two serotypes did not cluster by serotype. A total of 179 core genes did not show any particular pattern of clustering by serotype and another 169 core genes had identical sequences among all 90 isolates. Among trees constructed for the 19 AMR genes that were found in at least three genomes, only the tree for the core gene *aac (6′)-Iaa*, which encodes an enzyme that acetylates aminoglycosides, displayed clustering by serotype, supported by high bootstrap values (Additional file 2: Figure S4).

### Homologous recombination

The number of putative genome-wide recombination events detected by Gubbins differed among serotypes with 26, 1, and 0 intra-serotype recombination events identified in AMR *S.* Typhimurium, *S.* Newport, and *S.* Dublin, respectively (Additional file 1: Table S11). In addition to the genome-wide recombination analysis, homologous recombination was examined for each of the orthologous genes (i.e., both core and accessory orthologous genes) using the PHI test and the program RDP4. A total of 42 genes showed strong evidence for recombination, defined as detection of a recombination signal with PHI as well as at least 4 of the 7 methods included in RDP4 (Table 2). These 42 genes included two core genes, one encoding a major facilitator superfamily (MFS) transporter and one encoding an oxidoreductase FeS-binding subunit. The remaining 40 genes were accessory genes, including 35 serotype-associated genes (Table 2) with 17 of these 35 genes clearly annotated as encoding prophage-relevant proteins, such as phage tail proteins, capsid proteins, protein NinG, baseplate assembly protein, and recombinases. The 35 serotype-associated genes with evidence for homologous recombination also included genes annotated as having functions related to conjugal transfer (e.g., the conjugal transfer protein TrbC) and DNA methylation (e.g., methyltransferase, DNA methylase).

**Table 2:** Orthologous genes with evidence of homologous recombination detected by both PHI and RDP4

Gene	No. of taxa^a^	Function^b^	Serotype-associated^c^	PHI *p* value	RDP^d^	GENECONV^d^	Bootscan^d^	Maxchi^d^	Chimaera^d^	SiSscan^d^	3Seq^d^
Cluster_25	90	oxidoreductase FeS-binding subunit	N	0.004813				3.74E-03	2.87E-02	4.17E-08	0.03547
Cluster_32	90	MFS transporter	N	< 0.0005	2.75E-05		3.02E-02	6.11E-10		2.20E-22	3.23E-07
Cluster_52	64	phage tail protein	N	< 0.0005	7.38E-17	5.85E-18	3.99E-18	6.09E-12	9.23E-13	3.88E-15	2.81E-24
Cluster_3844	64	phage tail protein	N	< 0.0005	9.42E-10	3.59E-07	2.92E-06	1.13E-11	3.03E-13	1.04E-15	7.99E-18
Cluster_3925	70	phage tail protein	N	0.004785	2.93E-03			5.82E-10	3.01E-07	3.85E-23	7.85E-05
Cluster_4291	57	phage tail protein I	Y	< 0.0005			3.10E-03	2.07E-07	3.77E-05		3.30E-05
Cluster_4292	57	recombinase	Y	0.01689	4.56E-03	8.03E-03	4.72E-03	6.89E-06	1.32E-06	1.01E-04	2.34E-06
Cluster_4306	52	mercuric transport protein periplasmic component	N	0.007786		1.51E-08		6.83E-05	8.67E-04		3.06E-03
Cluster_4307	52	mercuric transport protein	N	0.002263	3.91E-07	7.08E-06	2.05E-07	5.77E-05	2.23E-04	1.57E-09	1.01E-06
Cluster_4308	53	portal protein	Y	< 0.0005	2.37E-14	9.40E-09	2.38E-14	3.37E-14			2.66E-27
Cluster_4309	53	hypothetical protein	Y	< 0.0005	1.72E-05	2.65E-03		1.63E-12	9.43E-08		4.90E-06
Cluster_4310	53	DNA transfer protein	Y	0.002031	1.30E-03	7.12E-04		6.10E-08	2.54E-04	4.43E-05	1.73E-04
Cluster_4343	53	RecBCD nuclease inhibitor	Y	< 0.0005	3.10E-05	2.10E-05	3.09E-06	4.82E-08	2.58E-06	1.16E-08	1.51E-06
Cluster_4344	53	recombinase	Y	< 0.0005		1.05E-02		3.44E-02	2.66E-02		2.01E-04
Cluster_4345	53	hypothetical protein	Y	< 0.0005		5.39E-04	5.88E-04	3.18E-07	2.55E-03	2.45E-09	4.45E-03
Cluster_4355	52	phage tail protein	Y	< 0.0005	1.13E-09	7.64E-04	2.42E-06	3.57E-11	1.02E-03		3.51E-02
Cluster_4361	48	peptidase	Y	< 0.0005	3.81E-09		3.92E-12	4.40E-04	3.19E-05		1.13E-07
Cluster_4432	48	terminase	Y	< 0.0005	2.89E-11	3.68E-10	2.02E-09	1.67E-17	7.22E-08	1.55E-42	4.83E-46
Cluster_4484	49	endonuclease	Y	< 0.0005	1.81E-17	2.25E-18	3.38E-14	2.63E-03	1.42E-03	8.97E-35	1.08E-36
Cluster_4488	40	phage tail tape measure protein	Y	< 0.0005	2.99E-02	3.41E-03	1.81E-04	1.26E-04	6.51E-03	2.38E-17	1.73E-12
Cluster_4533	47	phage portal protein	Y	< 0.0005	2.78E-10	6.75E-10	5.49E-09	5.22E-10	7.45E-04	9.57E-18	9.99E-18
Cluster_4534	47	capsid protein	Y	0.01974				9.04E-09	3.53E-03	4.91E-09	2.78E-03
Cluster_4543	47	baseplate assembly protein	Y	< 0.0005	2.80E-07	6.92E-18	4.96E-03	3.08E-19	5.29E-09	5.49E-03	1.26E-23
Cluster_4548	47	phage tail tape measure protein	Y	0.01543				3.98E-03	3.09E-03	4.16E-04	1.25E-02
Cluster_4549	47	phage tail protein	Y	0.008323	3.81E-84	3.34E-76	4.42E-76	9.86E-40	1.45E-41	6.19E-30	4.44E-16
Cluster_4562	46	acyltransferase	Y	< 0.0005		1.26E-06	3.40E-09	8.88E-09	4.05E-05	1.26E-11	4.13E-06
Cluster_4594	44	coat protein	Y	< 0.0005		8.20E-94	2.62E-96	2.28E-24	1.67E-24	3.81E-31	2.22E-15
Cluster_4595	44	endorhamnosidase	Y	< 0.0005	5.09E-12	2.93E-10	6.52E-12	4.87E-11	4.76E-12	5.27E-11	7.77E-15
Cluster_4658	31	hypothetical protein	Y	< 0.0005	2.26E-06	2.30E-03		5.76E-04	1.95E-03		9.80E-04
Cluster_4805	37	DNA-invertase	N	< 0.0005	2.98E-41	3.16E-35	5.25E-38	8.71E-15	2.73E-11	1.33E-12	8.88E-16
Cluster_4879	31	DNA transfer protein	Y	0.001663	8.93E-11	2.59E-05	2.43E-06	2.58E-13	2.32E-02	2.16E-09	1.82E-04
Cluster_5031	31	protein NinG	Y	< 0.0005	2.85E-08	3.32E-09		8.24E-08	4.16E-08	1.95E-10	1.21E-09
Cluster_5479	19	resolvase	Y	< 0.0005	3.90E-09	8.64E-07	4.57E-08	5.37E-10	9.25E-09	9.55E-07	6.43E-13
Cluster_5556	17	replication of DNA	Y	< 0.0005		6.88E-30	1.04E-43	9.83E-28		4.13E-29	7.14E-79
Cluster_5572	16	DNA methylase	Y	< 0.0005	4.00E-03	1.32E-03		9.25E-04	7.54E-05	3.24E-05	1.52E-03
Cluster_5598	15	methyltransferase	Y	0.00691	1.70E-02		5.20E-03	2.55E-05			6.62E-05
Cluster_5601	15	relaxase NikB	Y	< 0.0005	1.36E-08	3.65E-05	1.39E-08	9.80E-10			2.41E-15
Cluster_5602	15	conjugal transfer protein TrbC	Y	0.02477			6.49E-03	3.23E-04	4.18E-03		2.77E-02
Cluster_5607	15	hypothetical protein	Y	< 0.0005	3.06E-19	8.34E-20	3.60E-21	1.58E-17	2.82E-19	7.02E-17	1.11E-15
Cluster_5609	15	conjugal transfer protein	Y	< 0.0005		1.98E-02	6.21E-04	2.37E-04			1.92E-05
Cluster_5640	15	hypothetical protein	Y	0.005552	3.33E-02	5.88E-03	3.31E-04	1.36E-02	6.51E-03	1.18E-04	5.09E-04
Cluster_6042	5	oxaloacetate decarboxylase	N	< 0.0005		6.88E-03	2.66E-02	9.52E-05			1.27E-07

^a^Number of isolates with the corresponding cluster gene

^b^Function was determined by NCBI Prokaryotic Genome Annotation Pipeline

^c^Y: serotype-clustered core genes (i.e., showed clustering by serotype in the gene tree), or serotype-associated accessory genes (i.e., significantly over- or under- represented in one serotype)

*N* not serotype-clustered core genes nor serotype-associated accessory genes

^d^p value of the statistic test; only significant *p*-values (< 0.05) are shown

### Positive selection

A total of 41 genes (11 core genes and 30 accessory genes) showed evidence for diversifying selection across all serotypes, as detected by the site model in PAML (FDR <  0.05) (Additional file 1: Table S12); none of these 41 genes showed evidence for recombination. Eight of the 11 core genes with evidence for positive selection were serotype-clustered (defined here as showing clustering by serotype in the gene tree topology), and 20 of the 30 accessory genes with evidence for positive selection were serotype-associated (defined here as being significantly over- or under- represented in one serotype). The 41 genes with evidence for diversifying selection across all serotypes included 13 genes encoding cell surface proteins (Additional file 1: Table S12). These proteins included the cell envelope integrity protein TolA, the outer membrane protein assembly factor BamA, the conjugal transfer protein TraH, the type-IV secretion system protein TraC, different permeases and transporters, and fimbriae, pili, flagella-related proteins as well as porins and the phosphoporin PhoE. Additional genes that showed evidence for diversifying selection across all serotypes included three genes encoding proteins secreted by type VI secretion system as well as the genes encoding the virulence factor YopJ, a colicin-like toxin, and a chloramphenicol efflux MFS transporter (Additional file 1: Table S12).

Tests for diversifying selection within a given serotype identified 18 genes with evidence for positive selection in AMR *S.* Typhimurium (FDR <  0.05). Genes encoding a phage tail protein, an anion permease, and a flagellin showed evidence for strong positive selection, indicated by an overall dN/dS (ω) value > 500 (Additional file 1: Table S12). In addition, about half of the sites in *pilJ*, which encodes a type IV pilus biogenesis protein, showed significant evidence for diversifying selection (frequency *p* = 47.14%, ω = 22.43) (Additional file 1: Table S12). Diversifying selection within AMR *S.* Newport was observed in 8 genes (FDR < 0.05); the strongest selection pressure was found in the core gene encoding a DNA-binding transcriptional regulator, with ω ~∞, *p* = 4.00% (Additional file 1: Table S12). No genes showed evidence for diversifying selection within AMR *S.* Dublin.

To identify whether directional selection has played a role during the divergence of the three serotypes, 2381 serotype-clustered core genes were tested using the branch-site model in PAML. These analyses identified 5, 8 and 7 serotype-associated core genes with evidence for directional selection in AMR *S.* Dublin, *S.* Newport and *S.* Typhimurium ancestral branches, respectively (Table 3). The ω values for genes undergoing directional selection were generally high (Table 3). The 5 genes with evidence for directional selection on the AMR *S.* Dublin branch (Table 3), included the genes encoding the flagellar M-ring protein FliF, and the fimbrial protein StiA. The 8 genes with evidence for directional selection on the AMR *S.* Newport branch (Table 3) included *invA* (encoding the invasion protein facilitating bacterial host cell invasion) and the gene encoding the outer membrane protein assembly factor BamA (Table 3). Directional selection on the AMR *S.* Typhimurium branch was detected in 7 genes, including genes encoding a porin, a E3 ubiquitin--protein ligase, and a D-serine/D-alanine/glycine transporter (Table 3). The gene encoding an autotransporter outer membrane beta-barrel domain-containing protein showed evidence for directional selection in all three serotype ancestral branches (Table 3) and also showed evidence for diversifying selection across all serotypes (Additional file 1: Table S12).

**Table 3:** Serotype-associated core genes undergoing positive selection specifically on AMR *S.* Dublin, *S.* Newport or *S.* Typhimurium ancestral branches

Gene	Ln L MA1^a^	Ln L MA^b^	*p* value^c^	FDR^d^	Function^e^	ω^f^	proportion of sites under pos. Selection(%)^g^
*S.* Dublin branch							
Cluster_1190	− 7073.691	− 7048.222	0.000	0.000	autotransporter outer membrane beta-barrel domain-containing protein	569.327	5.984
Cluster_1721	− 1052.362	− 1038.860	0.000	0.000	dipeptidase E	999.000	1.364
Cluster_1900	−2015.241	−2004.734	0.000	0.002	bifunctional indole-3-glycerol phosphate synthase/phosphoribosylanthranilate isomerase	999.000	0.526
Cluster_2035	− 2457.111	− 2446.938	0.000	0.002	flagellar M-ring protein FliF	999.000	0.482
Cluster_3324	−778.864	− 771.694	0.000	0.032	fimbrial protein StiA	999.000	0.804
S. Newport branch							
Cluster_831	− 1041.959	− 1024.731	0.000	0.000	ABC transporter ATP-binding protein	999.000	3.724
Cluster_875	−844.370	− 826.859	0.000	0.000	CaiF/GrlA family transcriptional regulator	999.000	4.986
Cluster_1190	− 7071.499	− 7051.968	0.000	0.000	autotransporter outer membrane beta-barrel domain-containing protein	999.000	6.661
Cluster_1461	− 4112.903	− 4103.963	0.000	0.003	2-oxoglutarate dehydrogenase E1 component	999.000	0.113
Cluster_1538	− 2076.594	− 2066.164	0.000	0.001	invasin	999.000	0.295
Cluster_1678	−2008.961	−2001.754	0.000	0.019	hypothetical protein	204.362	0.829
Cluster_3346	− 1744.612	− 1726.105	0.000	0.000	hypothetical protein	999.000	11.655
Cluster_3368	− 4010.845	− 3976.156	0.000	0.000	outer membrane protein assembly factor BamA	836.419	3.378
S. Typhimurium branch							
Cluster_476	−1969.539	− 1933.139	0.000	0.000	porin	859.863	7.172
Cluster_1190	− 7076.553	−7037.396	0.000	0.000	autotransporter outer membrane beta-barrel domain-containing protein	252.954	7.072
Cluster_1216	− 2790.059	− 2781.805	0.000	0.008	maltodextrin glucosidase	999.000	0.764
Cluster_1756	− 2830.518	− 2823.397	0.000	0.024	glutamine--fructose-6-phosphate aminotransferase	828.247	0.394
Cluster_2234	−1998.702	−1990.410	0.000	0.008	tryptophan permease	999.000	1.035
Cluster_2311	− 3370.510	− 3358.161	0.000	0.000	E3 ubiquitin--protein ligase	171.701	1.404
Cluster_2757	− 2077.394	− 2068.717	0.000	0.008	D-serine/D-alanine/glycine transporter	106.317	1.691

^a^Ln L MA1; Lognormal likelihood score for the null hypothesis that sites evolved following neutral model in the serotype ancestral branch

^b^Ln L MA; Lognormal likelihood score for the alternative hypothesis that sites evolved under positive selection in the serotype ancestral branch

^c^The test statistic was calculated as 2[(−Ln L MA1) - (−Ln L MA)]

^d^This column indicates the *p*-value after a FDR (False Discovery Rate) correction was carried out to correct for multiple comparison

^e^“Function” represents the gene function provided by the NCBI Prokaryotic Genome Annotation Pipeline

^f^ω, average dN/dS value for codon sites under positive selection. dN is the number of nonsynonymous changes divided by the number of nonsynonymous sites. dS is the number of synonymous changes divided by the number of synonymous sites. ω = 999.000 represent infinite values as dS = 0

^g^proportion of codon sites under positive selection

## Discussion

While a number of *S. enterica* serotypes include AMR strains and lineages, AMR strains representing serotypes Dublin, Newport, and Typhimurium are of particular public health importance and represent a range of host specificity categories, providing a model for developing a better understanding of the evolution and population genetics of AMR *S. enterica*. Through in-depth analysis of 90 isolates representing AMR *S.* Dublin, *S.* Newport and *S.* Typhimurium from humans and bovines from Washington state and New York state, we demonstrated that (i) AMR *S.* Dublin, *S.* Newport and *S.* Typhimurium exhibited distinct genomic characteristics and evolutionary patterns; (ii) genome content of AMR *S. enterica*, including AMR genes, was strongly associated with serotype; (iii) positive selection mainly targeted genes encoding cell surface proteins and genes likely to function in virulence and antimicrobial resistance, while homologous recombination mainly acted on prophage-associated genes. Overall, our data suggest that evolution of AMR characteristics in *S. enterica* shows serotype-specific patterns and may involve positive selection in antimicrobial resistance-related genes, in addition to acquisition of AMR genes through horizontal gene transfer.

### AMR *S.* Dublin, *S.* Newport and *S.* typhimurium display distinct genomic characteristics and evolutionary patterns

AMR *S.* Dublin isolates included in this study displayed a relatively large core genome and small pan genome, showed low diversity of AMR genes (i.e., presence of a few distinct AMR genes), and encoded a few prophages including Salmon_ST64B, Entero_Fels_2 and Entero_P22, which appeared to be associated with AMR *S.* Dublin isolates. These genomic characteristics might be explained by the relatively small *Ne*, a recent population bottleneck, clonal population structure (i.e., infrequent intra-serotype homologous recombination), and limited diversifying selection observed here in AMR *S.* Dublin. This observation is consistent with the evolution being mainly driven by genetic drift, coupled with negative selection in bacteria with small *Ne* [12, 20]. Compared to AMR *S.* Newport and *S.* Typhimurium, *S.* Dublin genomes contained significantly more pseudogenes, and more orthologous genes and gene ontologies specifically absent from this serotype, suggesting genome decay, which are documented characteristics in host-adapted pathogens [12, 14, 35]. The functions of genes and gene ontologies specifically absent in AMR *S.* Dublin (e.g., arginine:ornithine antiporter activity, L-arginine transport, UDP-galactopyranose mutase activity, teichoic acid transport activity, ribulokinase activity) may not be required for growth and survival in the host niches typically occupied by *S.* Dublin. For example, a previous study reported that the arginine-ornithine antiporter is crucial for supplying external arginine as substrate to the Arginine Deiminase System (ADS), which contributes to acid resistance through production of ammonia. Consequently, for many pathogens the ADS system has been linked to virulence and fitness in the host [36]. Specifically, ADS-mediated production of ammonia has been shown to significantly prolong the intracellular survival of different bacteria, including *Staphylococcus epidermidis* and *Streptococcus suis* [36, 37]. As *S.* Dublin seems to be adapted to cattle, which have a rumen pH (ranging between 5.7 and 7.3) [38] much higher than that of human stomach lumen (1.5 to 3.5) [39], acid tolerance may be less important in *S.* Dublin, resulting in the loss of genes participating in the ADS-dependent arginine catabolism. In addition, L-ribulokinase participates in one of the two L-arabinose catabolism pathways to generate L-ribulose 5-phosphate [40]. As L-arabinose has been shown to regulate virulence gene expression in *S. enterica* [41], loss of one pathway of L-arabinose may affect virulence gene expression in *S.* Dublin.

Similar to AMR *S.* Dublin, AMR *S.* Newport genomes displayed a relatively low level of diversity, which might be a result of the likely-emergence of antimicrobial resistance in one single lineage, Lineage IIC, with all isolates tested here classified into this lineage. Also, the low genomic diversity may be due to a recent population size reduction, a relatively clonal population structure, and the low frequency of positive selection detected in AMR *S.* Newport in this study. In addition, geographic barriers might contribute to the low level of diversity observed in our data set; this hypothesis is supported by the association between geographic location and different Newport lineages observed in previous MLST and whole genome sequencing (WGS) studies [2, 18, 42, 43]. The GO terms “Type I site-specific deoxyribonuclease complex and activity”, “glucosyltransferase activity”, “protein N-linked glycosylation via asparagine”, and “antimonite transport” were specifically present in AMR *S.* Newport. These GO terms may be particularly important for the growth and survival of *S.* Newport in diverse hosts and environments. For example, restriction enzymes are the primary bacterial defense against lytic phages. Therefore, the presence of type I site-specific deoxyribonuclease complex and activity specifically in AMR *S.* Newport may represent a specific defense system against lytic phages in this serotype. In addition, glycans resulting from asparagine-linked glycosylation play a crucial role in various biological processes, such as protein folding, cellular targeting and motility, and immune response, in all three domains of life [44]. Hence, having the N-linked glycosylation via asparagine function may increase the fitness of *S.* Newport in host niches. Another interesting GO term specifically detected in AMR *S.* Newport is the biological process of antimonite transport. Antimonite is the salt of antimony (Sb(III)) and is toxic to cells [45]. Existence of antimonite transport in AMR *S.* Newport suggest that AMR *S*. Newport may have the ability to pump antimonite out of a cell, consequently conferring antimonite resistance. Interestingly, it has been suggested that microorganisms with multiple heavy metal resistances (including antimonite resistance) may also show resistance to some antibiotics due to co-location of heavy metal and antimicrobial resistance genes on the same mobile elements [46–48].

AMR *S.* Typhimurium exhibited a high level of genetic diversity, indicated by a relative open pan genome, a small core genome, a high diversity of AMR genes, and large variation in the number of AMR genes and prophage genes per genome. The high genetic diversity of AMR *S*. Typhimurium might result from its large *Ne*, relatively stable *Ne* over time, panmictic population structure (i.e., frequent homologous recombination), and relatively frequent positive selection observed in this study. These observations are consistent with the hypothesis that in large populations, selection overpowers genetic drift [20]. In addition, since we specifically selected AMR isolates for this study, the large pan genome size and diverse genome content of AMR *S.* Typhimurium may be associated with the emergence of antimicrobial resistance in multiple *S.* Typhimurium lineages, unlike the other broad-host serotype *S.* Newport, where antimicrobial resistance seems to have emerged in a single lineage (IIC). Specifically, at least two MDR *S.* Typhimurium groups have emerged in the past decades, DT 104 and DT 193 [27]. Based on the comparison with the MLST sequence type for DT 104 and DT 193 reference isolates, our dataset appears to include isolates belonging to at least two MDR Typhimurium lineages. A total of 22 GO and EC terms (e.g., inositol catabolic process and sphingolipid metabolic processes, which were shown to contribute to pathogenesis in mammalian hosts [49, 50]), were found to be specifically present in AMR *S.* Typhimurium. The functions associated with these gene ontologies might help *S.* Typhimurium to successfully compete in different niches and hosts. Notably, the GO term “regulation of autophagy” was also found to be specifically present in AMR *S.* Typhimurium. Autophagy is induced in the host to combat infection with various pathogenic bacteria, in which a double-membrane structure – autophagosome – engulfs invading pathogens and brings them to the lysosome for degradation [51, 52]. However, *S*. Typhimurium has been shown to exploit eukaryotic autophagy machinery during its intracellular life style in the host by inducing autophagic response to enter the host cell and suppressing autophagy within 3 h of infection [53]; our data suggest that *Salmonella* serotypes may differ in their ability to suppress autophagy as genes involved in regulation of autophagy function were only found in *S.* Typhimurium.

### Genome content of AMR *S. enterica*, including some AMR genes, is strongly associated with serotype

Even though serotypes within a bacterial species are defined based on cell surface antigens, genome content is typically correlated to serotype [10, 11]. Our data showed that about half of the accessory genes were “serotype-associated” (i.e., significantly over- or under-represented in a given serotype) and the majority of core genes were “serotype-clustered” (i.e., phylogenetic clustering by serotype). The strong association between AMR *S. enterica* genome content and serotype could be explained by the periods of elevated serotype diversification (e.g., due to host immune response) followed by long time of relatively lower but constant diversification observed in *Salmonella* [54].

Consistent with a previous analysis of the genomes studied here [7], different AMR genes were found to be associated with *Salmonella* serotypes. The association between AMR genes and AMR *S.* Typhimurium was especially strong, indicated by 5 AMR genes (*blaCARB*, *sulI, aadA*, *tetRG*, and *tetG*) significantly enriched and 6 AMR genes (*blaCMY*, *sulII*, *tetA*, *tetR*, *strA*, and *strB*) significantly under-represented in this serotype. *blaCARB*, *sulI*, *aadA*, *tetRG*, and *tetG* have been previously reported to be significantly associated with plasmid replicon IncFII(S) [7]; IncFII plasmids carrying AMR genes have previously been reported for some *S.* Typhimurium strains [55]. *blaCMY*, *sulII*, *tetA*, *tetR*, *strA*, and *strB* were previously reported to be significantly associated with the plasmid replicon IncA/C2 [7]. These resistance genes had previously been detected on a IncA/C2 plasmid in *S.* Newport [56] and were found in all *S.* Newport isolates analyzed here. Interestingly, the only AMR gene present in all 90 genomes, *aac (6′)-Iaa*, displayed a robust clustering by serotypes in the gene tree. AAC (6′)-Iaa is a chromosomal-encoded aminoglycoside acetyltransferase, which effectively acetylates tobramycin, kanamycin, and amikacin [57]. This result indicates that *aac (6′)-Iaa* might have been introduced into an ancestral strain of *S.* Dublin, *S.* Newport and *S.* Typhimurium before serotype diversification and subsequently transmitted vertically, through chromosome replication.

### While homologous recombination mainly acts on prophage-associated genes, positive selection mainly targets genes encoding cell surface proteins and genes likely to function in virulence and antimicrobial resistance in AMR *S. enterica*

Homologous recombination and positive selection have been shown to play critical roles in the evolution of bacteria including *Salmonella* [15, 58, 59]. In this study of AMR *Salmonella*, a number of genes showed evidence for homologous recombination or positive selection. Most of these genes were serotype-associated, suggesting the contribution of homologous recombination and positive selection in serotype diversification.

Most of the serotype-associated accessory genes showing evidence for homologous recombination encode prophage-associated proteins, such as phage tail proteins, capsid proteins, the protein NinG, a baseplate assembly protein, and recombinases. This result supports phage-mediated homologous recombination as one important mechanism in the remodeling of the bacterial genome [60]. Moreover, this result suggests that detectable homologous recombination is limited outside of prophage sequences. This could be due to *Salmonella*’s limited ability to acquire foreign DNA through transformation [61] or the low chromosomal diversity within serotype, which renders few nucleotide polymorphisms within recombinant fragments [60].

Congruent with previous observations that cell surface proteins are main targets of positive selection in both eukaryotes and prokaryotes [15, 62–66], most genes showing evidence for positive selection in this study encode cell surface proteins, such as outer membrane proteins, transporters, permeases, porins, cell surface appendages. Notably, a number of the proteins found here to show evidence for positive selection have reported or plausible functions in virulence, including genes encoding fimbriae, pili, flagella-related proteins [67, 68]. In addition, an autotransporter outer membrane beta-barrel domain-containing protein and the outer membrane protein assembly factor BamA, which both participate in the beta-barrel assembly, showed evidence for positive selection and have been proposed to play direct and indirect roles in virulence in Gram-negative bacteria [69]. Genes where positive selection may contribute to antimicrobial resistance include those encoding TraC and TraH, a chloramphenicol efflux MFS transporter, and a number of porins, including PhoE. Briefly, the VirB5-like TraC protein and VirB8-like TraH protein, which are two type-IV secretion system proteins, are virulence factors that may be targeted by antibiotics [70–72]. The chloramphenicol efflux MFS transporter functions as an export pump for the antimicrobial chloramphenicol [73]. Porins such as OmpF, OmpC, and PhoE, play an important role in allowing influx of β-lactam antibiotics in Gram-negative bacteria, which is important to allow these and potentially other antibiotics to access their targets [74]. As previous studies have indicated that specific mutations in porins may contribute to increased resistance to β-lactam antibiotics [15], our findings suggest that positive selection in porins could contribute to AMR phenotypes and adaptation of *Salmonella* to antibiotics. Porins also may play a role in virulence and have been reported to inhibit leukocyte phagocytosis by activating the adenylate cyclase system in *S.* Typhimurium [75].

Genes that were found to have evidence for positive selection, but do not encode cell surface proteins, include genes encoding proteins that appear to contribute to bacterial virulence (e.g., E3 ubiquitin-protein ligase, effector protein YopJ, invasion, D-alanine transporter) or bacteria-bacteria interactions (e.g., type IV secretion protein Rhs, and colicin) [76–79]. Interestingly, two of these proteins (E3 ubiquitin-protein ligase and D-alanine transporter) were also identified as being gradually lost in *S.* Cerro, which appears to show reduced human virulence [80]. In addition, some prophage-related genes, such as phage tail gene and baseplate gene, showed evidence for positive selection. Most interestingly, diversifying selection across all three serotype was detected in a gene encoding a 23S rRNA (guanine (745)-N (1))-methyltransferase. This methyltransferase methylates the 23S rRNA of Gram-negative bacteria at nucleotide G745, which is located at the peptide exit channel of the ribosome. This same site has been shown to be the binding site of macrolide, lincosamide, and streptogramin B antibiotics [81]. While it is not clear whether the methylation of G745 confers resistance to these antibiotics, mutation in this methyltransferase gene have been shown to increase resistance of *Escherichia coli* to the ribosome binding antibiotic viomycin [82]. Hence, positive selection detected in the gene encoding a 23S rRNA (guanine (745)-N (1))-methyltransferase might contribute to an increased resistance of *S. enterica* against some ribosome-targeted antibiotics. Overall, these findings suggest that positive selection may not only contribute to adaptation to stresses encountered in hosts and environmental niches, but also may play a role in the evolution of antimicrobial resistance.

## Conclusions

By performing a comprehensive genome-wide analysis, we show that antimicrobial-resistant *S.* Dublin, *S.* Newport and *S.* Typhimurium exhibited distinct evolutionary patterns and genomic characteristics. AMR *S.* Typhimurium showed a large and diverse genome and frequent positive selection and homologous recombination, consistent with multiple emergence events of AMR strains and/or effective dispersal and ecological success of this serotype in different niches. AMR *S.* Dublin showed evidence for experiencing a recent population bottleneck (similar to *Yersinia pestis* [83] and *Mycobacterium tuberculosis* [84]) and genome decay, which are consistent with its narrow host range. While our data suggest that homologous recombination and positive selection drive the evolution in some serotype-associated genes, suggesting a critical role of these evolutionary mechanisms in serotype diversification of *S. enterica*, this hypothesis warrants further investigation since only AMR isolates were included in this study. Importantly, a focus on analysis of AMR *Salmonella* isolates supports the importance of a range of mechanisms in the evolution of antimicrobial resistance and the emergence of AMR lineages, in addition to horizontal transfer of well characterized antimicrobial resistance genes (e.g., genes encoding beta lactamases). For example, while positive selection in target proteins of antibiotics (e.g., gyrase) is well documented [85], our study suggests that positive selection in a wider range of genes, including genes encoding target modification enzymes (e.g., 23S methyltranferases) and genes encoding proteins that facilitate target access (e.g., porins), may contribute to the evolution of antimicrobial resistance. Future efforts on understanding the evolution, spread, and maintenance of antimicrobial resistance in additional *Salmonella* serotypes and other human and zoonotic pathogens with different host ranges and geographical locations are thus needed to characterize a variety of evolutionary mechanisms (e.g., horizontal gene transfer, mutations, positive selection), including their relative roles and dependencies. For example, some horizontally transferred plasmids may lead to intermediate- or low-level resistance that is not clinically relevant but enhances the risk of subsequent emergence of fully resistant subtypes via selection of mutations that lead to high-level resistance, as recently proposed for quinolone resistance [86]. In addition, positive selection in some genes may represent compensatory mutations that facilitate the fixation of AMR genes in populations [87]. While increasing availability of whole genome sequence data will facilitate more in-depth evolutionary studies of complex mechanisms underpinning the emergence and spread of antimicrobial resistance and AMR lineages, these studies will need to be supplemented by functional studies that define the phenotypic impact of gene acquisitions and mutations hypothesized to be linked to antimicrobial resistance. Future large WGS data sets as well as the data set analyzed here also provide a future opportunity to explore associations between geographical locations and host species and evolutionary patterns of interest, including AMR acquisition.

## Methods

### Isolation selection

A previously reported set of genome sequences for 90 *S. enterica* isolates [7] collected from the stool samples of human patients presenting clinical signs of salmonellosis (*n* = 49) and the fecal samples of bovine (*n* = 41) between 2008 and 2012 was used for this study, 45 of which were from New York state and 45 were from Washington state (Additional file 1: Table S3). Those isolates represent one of the three serotypes of interest (Dublin (*n* = 21), Newport (*n* = 32) and Typhimurium (*n* = 37)), as determined using the White-Kauffmann-Le Minor scheme [88] and had previously been tested to be resistant to at least one antimicrobial drug [7].

### Whole genome sequencing and genome assembly

Methods of whole genome sequencing and genome assembly were detailed in [7]. The sequence data has been deposited in the National Center for Biotechnology Information’s (NCBI) Sequence Read Archive (SRA) under accession number SRP068320. Assembled genomes have been deposited at NCBI DDBJ/ENA/GenBank under the accession numbers listed in Additional file 1: Table S3.

### Reference-free and reference-based variant calling, phylogenetic tree construction and recent population dynamics inference

The reference-free variant calling among all 90 genomes and phylogenetic tree construction followed the methods provided by [7]. To confirm the lineages of *S.* Newport isolates, genome assemblies of *S.* Newport str. CVM 19443 (GenBank Accession AHUB00000000), *S.* Newport str. CVM 19567 (AHTR00000000), *S.* Newport str. SL254 (CP001113), *S.* Newport str. SL317 (ABEW00000000) were downloaded from GenBank as reference genomes of *S.* Newport Lineage IIA, IIB, IIC, and III, respectively. An ML tree of 32 *S.* Newport genomes and 4 reference genomes was constructed following the same approach as for the ML tree of 90 genomes.

To maximize resolution, variant calling was performed within each serotype using the Cortex variant caller (cortex_var) [89] following the steps provided in [80]. For *S.* Typhimurium isolates, *S.* Typhimurium str. LT2 (RefSeq NC_003197.1) was used as a reference genome; for *S.* Newport isolates, *S.* Newport str. SL254 (GenBank Accession CP001113) was used as a reference; for *S.* Dublin isolates, the contigs of isolate BOV_DUBN_WA_10_R9_3233 in this study were used as a reference as detailed in [7]. A cleaning threshold inferred from the coverage distribution, a quality score threshold of 15, a cutting homopolymer threshold of 15, and a population filter to remove false calls were applied in running cortex_var. SNPs were filtered from other variants such as indels using vcftools version 0.1.5 [90], and SNPs in the recombination regions were filtered out using Gubbins version 1.4.2 [91].

The resulting high-quality SNPs were used to infer population dynamics for each serotype using BEAUti version 1.8.4 and BEAST version 1.8.4 [92]. The best nucleotide substitution models for SNPs within each serotype determined by MEGA7 [93] were specified in BEAUti version 1.8.4. The best model identified for *S.* Typhimurium was the general time reversible (GTR) model [94], while that for *S.* Newport and *S.* Dublin is the Kimura 2-parameter model [95]. An ascertainment bias correction was applied as SNPs were used [7] and base frequencies were estimated. The prior substitution rate for *S.* Typhimurium was set to 1 × 10^− 6^ /site/year [23]. *S.* Newport and *S.* Dublin were set to the average rate of bacteria (2.1 × 10^− 7^ /site/year) since the literature lacks estimations of substitution rates for either serotype. An MCMC algorithm was run for 100 million generations, with sampling every 10,000 generations, to estimates the posterior probability distributions of the genealogical and demographic parameters of a sample. Marginal likelihood estimations were computed by path sampling [96, 97] and stepping stone sampling using 100 steps with a chain length of 1 million generations, sampling every 1000 generations. For each serotype, combinations of either a (i) strict or (ii) lognormal relaxed molecular clock, and either a (i) coalescent constant size, (ii) exponent growth population size, (iii) Bayesian skygrid population or (iv) Bayesian skyline population were tested, and for each combination, three 100 million MCMC replicate runs were performed. Bayes factors, representing the ratio of the marginal likelihood of model, were compared among combination models. According to the Bayes factors associated with each molecular clock-population model combination, the best model for all three serotypes was the model of a relaxed molecular clock with a Bayesian skyline growth population. The log and trees files of converging individual replicate runs were combined in LogCombiner version 1.8.4 with a burn-in of 10,000,000 sampling every 100,000 states. The combined trees file was edited in FigTree version 1.4.2 with posterior probabilities being placed on the nodes. Effective sample sizes of run statistics examined by TRACER version 1.5 indicated convergence and adequate mixing of the Markov chains. Using TRACER version 1.5, the first 10% of each chain were discarded as burn-in and the Bayesian skyline plot indicating the population dynamics was reconstructed for each serotype.

### In silico AMR gene and prophage detection, and multi-locus sequence typing of *S.* Typhimurium

AMR genes in all 90 assembled genome sequences were determined by nucleotide BLAST version 2.4.0 [98] and the formatted ARG-ANNOT database included with SRST2 [99]. The results of AMR genes were previously reported by [7].

The putative prophage-associated proteins were detected based on the sequence similarity of the protein-coding gene predicted by Prodigal version 2.60 [100] to a curated prophage protein dataset. The dataset, containing 265,986 prophage protein sequences, was downloaded in January 2016 from the PHAST server [101]. These prophage proteins were clustered into 167,195 groups by using CD-HIT version 4.6.5 [102] with a sequence similarity threshold of 90% identity. The 536 groups related to integrases or transposases were removed and the remaining 166,659 groups served as the PPG dataset used in this study. Protein BLAST was performed using proteins predicted by Prodigal version 2.60 against the PPG dataset. Query proteins with significant similarities (identities > 80% and query coverage > 80%) were classified as putative prophage proteins and subsequently clustered into the most similar PPG, resulting in 732 PPGs across the 90 genomes.

To determine the sequence type of AMR *S.* Typhimurium isolates analyzed in this study, two reference strains of phage type DT 104 (GenBank Accession NC_022569, GCA_002034985), one reference strain of phage type DT 193 – str. SO4698–09 (NZ_LN999997), and all the 37 assembled Typhimurium genomes from our dataset were analyzed using the MLST online service provided by Center for Genomic Epidemiology (https://cge.cbs.dtu.dk/services/MLST/).

### Genome annotation, identification of orthologous genes, gene tree construction, and gene ontology and enzyme commission

Assembled genomes were annotated by the NCBI Prokaryotic Genome Annotation Pipeline [103]. OrthoMCL [104] was used to identify orthologous genes in the 90 annotated genomes. Amino acid sequences and nucleotide sequences of each ortholog were extracted from GeneBank using Genbank/EMBL to FASTA Conversion Tool (https://rocaplab.ocean.washington.edu/tools/genbank_to_fasta/). Amino acid sequences of each orthologous group were aligned using MUSCLE version 3.7 [105]. Amino acid sequence alignments were converted into nucleotide sequence alignments using PAL2NAL version 14 [106]. Gene trees of core orthologous genes and AMR genes were constructed with RAxML version 8.2.4 [107] under the general time-reversible model. All gene trees of orthologous genes were midpoint rooted and their branch lengths were removed in RStudio version 1.0.136 [108]. Customized Python scripts were used to detect clustering by serotypes in each gene tree. Core genes for which tree topology showed a clustering by serotype were defined as serotype-associated core genes. GO and EC terms were assigned to all 90 annotated genomes using Blast2GO v1.2.1 [109].

### Homologous recombination detection

The Recombination Detection Program (RDP) and Pairwise Homoplasy Index (PHI) test were used to detect homologous recombination in individual orthologous genes. Cleaned-up alignments in which identical sequences were removed were used. The PHI test was performed in SplitsTree4 version 4.14.2 [110]. Seven homologous recombination detection methods (RDP, BOOTSCAN, GENECONV, MAXCHI, CHIMAERA, SISCAN, and 3Seq) incorporated in RDP4 [111] were further employed using the default settings. To reduce false positives, only recombination events detected by at least four of the methods used in RDP4 [112, 113] and also by PHI test in orthologous genes were considered to be significant events. Genome-wide recombination within each serotype were inferred by Gubbins version 1.4.2 [91] on concatenated sequence alignment using reference-based assembled genomes by cortex_var.

### Positive selection detection

Nucleotide sequence alignments of protein coding genes containing isolates from all three serotypes and only isolates from each serotype were cleaned up by removing identical sequences. Alignments that had more than three non-identical sequences and contained at least one non-synonymous mutation were used for positive selection analyses using the PAML program version 4.8 [114]. Unrooted trees constructed by RAxML version 8.2.4 for each gene were used. Diversifying selection across codon sites among three serotypes and within each serotype was assessed using a likelihood ratio test (LRT) by comparing M1a model (nearly neutral) against M2a model [positive selection in a fraction of the sites (ω > 1)] (site model). Directional selection on specific ancestral branches to each of the three serotypes were assessed using LRT by comparing MA1 (positive selection model) against MA (neutral evolution model) (branch-site model) on core genes which showed clustering by serotype on the gene tree. Details of parameters used in each model were described in [115, 116]. The LRT statistic was calculated by 2 × ln (L_0_/L_1_), where L_0_ is the likelihood estimate for null model (neutral model) and L_1_ is the likelihood estimate for alternative model (positive selection model) which has more free parameters. The degree of freedom (df), which is calculated as the difference in the number of free parameters between the null and alternative models, was set to 2 for site model and 1 for site-branch model. Statistical significance was determined by approximating the test statistic to a χ2 distribution. A false discovery rate (FDR) correction was applied and genes with FDR < 0.05 were considered significant.

### Statistics

The presence/absence of orthologous gene content table was used to perform the rarefaction curve estimation of core genome size and accumulation curve of pan genome size (i.e., the sum of core genes and accessory genes) estimation for each serotype using an R software script provided by [117]. The procedure was repeated 100 times, randomizing the order of the genomes every time to obtain the mean core and pan genome size estimates and standard errors. Non-metric multidimensional scaling was employed to compare the dissimilarity of gene presence/absence pattern among serotypes based on Bray-Curtis distance using RStudio version 1.0.136 [108]. Shannon-wiener index was calculated, using the abundance of AMR genes among isolates within each serotype to indicate the AMR gene diversity. Over-representation and under-representation of orthologous genes, GO terms, AMR genes and PPG was assessed using Fisher’s exact tests in RStudio version 1.0.136 [108] by comparing the presence/absence of features in one serotype against the other two. The FDR procedure [118] was applied to correct for multiple testing. Items significantly over- or under-represented in one serotype were defined as the ones identified as significant in given comparisons relative to the other two serotypes (FDR < 0.05, odds ratio >6.71 or  < 0.15, respectively, for over- / under- represented) [119]. The accessory genes that were significantly over- or under- represented in one serotype were defined as serotype-associated accessory genes. Pseudogenes were extracted from GenBank files of each isolate and Kruskal-Wallis test [120] was carried out to determine if the numbers of pseudogenes differed significantly among the three serotypes.

## Additional files

Additional file 1: **Table S1.** Distribution of the orthologous genes identified among all 90 genomes. **Table S2.** The pan and core genome size of all three serotypes and each serotype. **Table S3.** Basic information of 90 AMR *S. enterica* isolates. **Table S4.** Distribution of the AMR genes identified among all 90 genomes. **Table S5.** Distribution of the PPG genes identified among all 90 genomes. **Table S6.** Orthologous genes significantly over- and under- represented in S. Dublin, S. Newport, and S. Typhimurium. **Table S7.** Distributionof the GO and EC terms identified among all 90 genomes. **Table S8.** GO terms and EC numbers significantly over- and under- represented in S. Dublin, S. Newport, and S. Typhimurium. **Table S9.** Prophages and number of corresponding PPG genes significantly over- and under- represented in S. Dublin, S. Newport, and S. Typhimurium. **Table S10.** MLST sequence type of S. Typhimurium isolates. **Table S11.** Isolates identified by Gubbins showing recombinant regions. **Table S12.** Genes undergoing positive selection across all serotypes, within S. Newport and S. Typhimurium detected by site model using PAML. (XLSX 3780 kb)

Additional file 2: **Figure S1.** The distribution of AMR gene numbers of AMR *S.* Dublin, AMR *S.* Newport, and AMR *S.* Typhimurium isolates. The AMR gene number of AMR *S.* Dublin is significantly different from that of AMR *S.* Newport and AMR *S.* Typhimurium. **Figure S2.** The distribution of PPG gene numbers of AMR *S.* Dublin, AMR *S.* Newport, and AMR *S.* Typhimurium isolates. **Figure S3.** The distribution of pseudogene numbers in *S.* Dublin, *S.* Newport, and *S.* Typhimurium. **Figure S4.** Gene tree of AAC (6′)-Iaa inferred by maximum likelihood method. Tree is rooted by midpoint. Bootstrap values > 70% are presented on the tree. *S.* Dublin is indicated by blue, *S.* Newport by blue, and *S.* Typhimurium by red. **Figure S5.** Maximum likelihood tree of AMR *S.* Newport isolates, and Lineage II (sub-lineages - IIA, IIB and IIC) and Lineage III reference isolates. Tree is rooted by midpoint. Bootstrap values of major clades are presented on the tree. Reference isolates are indicated by red. (DOCX 681 kb)

## Data Availability

The sequence data has been deposited in the National Center for Biotechnology Information’s (NCBI) Sequence Read Archive (SRA) under accession number SRP068320. Assembled genomes have been deposited at NCBI DDBJ/ENA/GenBank under the accession numbers listed in Additional file 1: Table S3.

## Abbreviations

ADS: Arginine Deiminase System
AMR: Antimicrobial-resistant
EC: Enzyme commission
FDR: False Discovery Rate
GO: Gene ontology
GTR: General time reversible
LRT: Likelihood ratio test
MCMC: Markov chain Monte Carlo
MDR: Multidrug-resistant
MFS: Major facilitator superfamily
ML: Maximum likelihood
MLST: Multilocus sequence typing
NCBI: National Center for Biotechnology Information
*Ne*: Effective population size
PBP: Penicillin-binding protein
PHI: Pairwise Homoplasy Index
PPG: Prophage protein group
RDP: Recombination Detection Program
SNP: Single nucleotide polymorphism
SRA: Sequence Read Archive
ST: Sequence type
WGS: Whole genome sequencing

## References

[1] Desai PT, Porwollik S, Long F, Cheng P, Wollam A, Clifton SW (2013). Evolutionary genomics of *Salmonella enterica* subspecies. MBio..

[2] Cao G, Meng J, Strain E, Stones R, Pettengill J, Zhao S (2013). Phylogenetics and differentiation of *Salmonella* Newport lineages by whole genome sequencing. PLoS One.

[3] Majowicz SE, Musto J, Scallan E, Angulo FJ, Kirk M, O'Brien SJ (2010). International collaboration on enteric disease “burden of illness” studies. The global burden of nontyphoidal *Salmonella* gastroenteritis. Clin Infect Dis.

[4] Scallan E, Hoekstra RM, Angulo FJ, Tauxe RV, Widdowson MA, Roy SL (2011). Foodborne illness acquired in the United States--major pathogens. Emerg Infect Dis.

[5] USDA (2015). *Salmonella* tops list of 15 Most costly pathogens.

[6] Hong S, Rovira A, Davies P, Ahlstrom C, Muellner P, Rendahl A (2016). Serotypes and antimicrobial resistance in *Salmonella enterica* recovered from clinical samples from cattle and swine in Minnesota, 2006 to 2015. PLoS One.

[7] Carroll LM, Wiedmann M, den Bakker H, Siler J, Warchocki S, Kent D (2017). Whole-genome sequencing of drug-resistant *Salmonella enterica* isolates from dairy cattle and humans in New York and Washington states reveals source and geographic associations. Appl Environ Microbiol.

[8] Eng SK, Pusparajah P, Ab Mutalib NS, Ser HL, Chan KG, Lee LH (2015). *Salmonella*: a review on pathogenesis, epidemiology and antibiotic resistance. Front Life Sci.

[9] Uzzau S, Brown DJ, Wallis T, Rubino S, Leori G, Bernard S (2000). Host adapted serotypes of *Salmonella enterica*. Epidemiol Infect.

[10] Jacobsen A, Hendriksen RS, Aaresturp FM, Ussery DW, Friis C (2011). The *Salmonella enterica* pan-genome. Microb Ecol.

[11] Zou QH, Li RQ, Liu GR, Liu SL (2016). Genotyping of *Salmonella* with lineage-specific genes: correlation with serotyping. Int J Infect Dis.

[12] Bobay LM, Ochman H (2017). The evolution of bacterial genome architecture. Front Genet.

[13] Bäumler AJ, Tsolis RM, Ficht TA, Adams LG (1998). Evolution of host adaptation in *Salmonella enterica*. Infect Immun.

[14] Langridge GC, Fookes M, Connor TR, Feltwell T, Feasey N, Parsons BN (2015). Patterns of genome evolution that have accompanied host adaptation in *Salmonella*. Proc Natl Acad Sci U S A.

[15] Soyer Y, Orsi RH, Rodriguez-Rivera LD, Sun Q, Wiedmann M (2009). Genome wide evolutionary analyses reveal serotype specific patterns of positive selection in selected *Salmonella* serotypes. BMC Evol Biol.

[16] Parkhill J, Dougan G, James KD, Thomson NR, Pickard D, Wain J (2001). Complete genome sequence of a multiple drug resistant *Salmonella enterica* serovar Typhi CT18. Nature..

[17] Chattopadhyay S, Paul S, Kisiela DI, Linardopoulou EV, Sokurenko EV (2012). Convergent molecular evolution of genomic cores in *Salmonella enterica* and *Escherichia coli*. J Bacteriol.

[18] Sangal V, Harbottle H, Mazzoni CJ, Helmuth R, Guerra B (2010). Evolution and population structure of *Salmonella enterica* serovar Newport. J Bacteriol.

[19] Didelot X, Achtman M, Parkhill J, Thomson NR, Falush D (2007). A bimodal pattern of relatedness between the *Salmonella* Paratyphi A and Typhi genomes: convergence or divergence by homologous recombination?. Genome Res.

[20] Bromham L, Penny D (2003). The modern molecular clock. Nat Rev Genet.

[21] Roumagnac P, Weill FX, Dolecek C, Baker S, Brisse S, Chinh NT (2006). Evolutionary history of *Salmonella* Typhi. Science..

[22] Hendriksen RS, Leekitcharoenphon P, Lukjancenko O, Lukwesa-Musyani C, Tambatamba B, Mwaba J (2015). Genomic signature of multidrug-resistant *Salmonella enterica* serovar Typhi isolates related to a massive outbreak in Zambia between 2010 and 2012. J Clin Microbiol.

[23] Hawkey J, Edwards DJ, Dimovski K, Hiley L, Billman-Jacobe H, Hogg G (2013). Evidence of microevolution of *Salmonella* typhimurium during a series of egg-associated outbreaks linked to a single chicken farm. BMC Genomics.

[24] Wang L, Cai X, Wu S, Bomjan R, Nakayasu ES, Händler K (2017). *invS* coordinates expression of *prgH* and *fimZ* and is required for invasion of epithelial cells *by Salmonella enterica* serovar typhimurium. J Bacteriol.

[25] Farzan A, Friendship RM, Poppe C, Martin L, Dewey CE, Funk J (2008). Molecular epidemiology and antimicrobial resistance of *Salmonella* typhimurium DT104 on Ontario swine farms. Can J Vet Res.

[26] Chiu CH, Su LH, Chu CH, Wang MH, Yeh CM, Weill FX, Chu C (2006). Detection of multidrug-resistant *Salmonella enterica* serovar typhimurium phage types DT102, DT104, and U302 by multiplex PCR. J Clin Microbiol.

[27] Gebreyes WA, Altier C (2002). Molecular characterization of multidrug-resistant *Salmonella enterica* subsp. *enterica* serovar typhimurium isolates from swine. J Clin Microbiol.

[28] Nuccio SP, Bäumler AJ (2014). Comparative analysis of *Salmonella* genomes identifies a metabolic network for escalating growth in the inflamed gut. MBio..

[29] Gupta A, Fontana J, Crowe C, Bolstorff B, Stout A, Duyne SV (2003). Emergence of multidrug-resistant *Salmonella enterica* serotype Newport infections resistant to expanded-spectrum cephalosporins in the United States. J Infect Dis.

[30] Akiba M, Nakaoka Y, Kida M, Ishioka Y, Sameshima T, Yoshii N (2007). Changes in antimicrobial susceptibility in a population of *Salmonella enterica* serovar Dublin isolated from cattle in Japan from 1976 to 2005. J Antimicrob Chemother.

[31] Harvey RR, Friedman CR, Crim SM, Judd M, Barrett KA, Tolar B (2017). Epidemiology of *Salmonella enterica* serotype Dublin infections among humans, United States, 1968–2013. Emerg Infect Diseases.

[32] Davis MA, Hancock DD, Besser TE, Daniels JB, Baker KN, Call DR (2007). Antimicrobial resistance in Salmonella enterica serovar Dublin isolates from beef and dairy sources. Vet Microbiol.

[33] Andino A, Hanning I (2015). *Salmonella enterica*: survival, colonization, and virulence differences among serovars. Sci World J.

[34] Sun S, Selmer M, Andersson DI (2014). Resistance to β-lactam antibiotics conferred by point mutations in penicillin-binding proteins PBP3, PBP4 and PBP6 in *Salmonella enterica*. PLoS One.

[35] Wren BW (2000). Microbial genome analysis: insights into virulence, host adaptation and evolution. Nature Rev Genet.

[36] Fulde M, Willenborg J, Huber C, Hitzmann A, Willms D, Seitz M (2014). The arginine-ornithine antiporter ArcD contributes to biological fitness of *Streptococcus suis*. Front Cell Infect Microbiol.

[37] Lindgren JK, Thomas VC, Olson ME, Chaudhari SS, Nuxoll AS, Schaeffer CR (2014). Arginine deiminase in *Staphylococcus epidermidis* functions to augment biofilm maturation through pH homeostasis. J Bacteriol.

[38] Hungate RE (1966). The rumen and its microbes.

[39] Marieb EN, Hoehn K. Human anatomy & physiology. San Francisco: Benjamin Cummings; 2010.

[40] Fraser-Reid BO, Tatsuta K, Thiem J, editors. Glycoscience: chemistry and chemical biology I–III. Berlin: Springer; 2012.

[41] López-Garrido J, Puerta-Fernández E, Cota I, Casadesús J (2015). Virulence Gene Regulation by L-arabinose in *Salmonella enterica*. Genetics..

[42] Sukhnanand S, Alcaine S, Warnick LD, Su WL, Hof J (2005). DNA sequence-based subtyping and evolutionary analysis of selected *Salmonella enterica* serotypes. J Clin Microbiol.

[43] Torpdahl M, Skov MN, Sandvang D, Baggesen DL (2005). Genotypic characterization of *Salmonella* by multilocus sequence typing, pulsed-field gel electrophoresis and amplified fragment length polymorphism. J Microbiol Methods.

[44] Larkin A, Imperiali B (2011). The expanding horizons of asparagine-linked glycosylation. Biochemistry..

[45] Hasgekar N, Beck JP, Dunkelberg H, Hirsch-Ernst KI, Gebel TW (2006). Influence of antimonite, selenite, and mercury on the toxicity of arsenite in primary rat hepatocytes. Biol Trace Elem Res.

[46] Farias P, Santo CE, Branco R, Francisco R, Santos S, Hansen L (2015). Natural hot spots for gain of multiple resistances: arsenic and antibiotic resistances in heterotrophic, aerobic bacteria from marine hydrothermal vent fields. Appl Environ Microbiol.

[47] Dib J, Motok J, Zenoff VF, Ordoñez O, Farías ME (2008). Occurrence of resistance to antibiotics, UV-B, and arsenic in bacteria isolated from extrme environments in high-altitude (above 4400 m) Andean wetlands. Curr Microbiol.

[48] De Souza MJ, Nair S, Bharathi PL, Chandramohan D (2006). Metal and antibiotic-resistance in psychrotrophic bacteria from Antarctic marine waters. Ecotoxicology..

[49] Pralhada Rao Raghavendra, Vaidyanathan Nanditha, Rengasamy Mathiyazhagan, Mammen Oommen Anup, Somaiya Neeti, Jagannath M. R. (2013). Sphingolipid Metabolic Pathway: An Overview of Major Roles Played in Human Diseases. Journal of Lipids.

[50] Xue C (2015). Finding the sweet spot: how human fungal pathogens acquire and turn the sugar inositol against their hosts. mBio..

[51] Randow F, Münz C (2012). Autophagy in the regulation of pathogen replication and adaptive immunity. Trends Immunol.

[52] Hong BY, Croxen MA, Marchiando AM, Ferreira RB, Cadwell K, Foster LJ (2014). Autophagy facilitates *Salmonella* replication in HeLa cells. MBio..

[53] Casanova James E. (2017). Bacterial Autophagy: Offense and Defense at the Host–Pathogen Interface. Cellular and Molecular Gastroenterology and Hepatology.

[54] Deng X, den Bakker HC, Hendriksen RS. Applied genomics of foodborne pathogens. Berlin: Springer; 2017.

[55] Beutlich J, Rodicio MR, Mendoza MC, García P, Kirchner M (2013). *Salmonella enterica* serovar typhimurium virulence-resistance plasmids derived from the pSLT carrying nonconventional class 1 integrons with *dfrA12* gene in their variable region and *sul3* in the 3′ conserved segment. Microb Drug Resist.

[56] Fricke WF, Welch TJ, McDermott PF, Mammel MK, LeClerc JE, White DG (2009). Comparative genomics of the IncA/C multidrug resistance plasmid family. J Bacteriol.

[57] Salipante SJ, Hall BG (2003). Determining the limits of the evolutionary potential of an antibiotic resistance gene. Mol Biol Evol.

[58] McClelland M, Sanderson KE, Spieth J, Clifton SW, Latreille P, Courtney L (2006). *Yersinia* YopJ acetylates and inhibits kinase activation by blocking phosphorylation. Science.

[59] Leekitcharoenphon P, Lukjancenko O, Friis C, Aarestrup FM, Ussery DW (2012). Genomic variation in *Salmonella enterica* core genes for epidemiological typing. BMC Genomics.

[60] Menouni R, Hutinet G, Petit MA, Ansaldi M (2015). Bacterial genome remodeling through bacteriophage recombination. FEMS Microbiol Lett.

[61] MacLachlan PR, Sanderson KE (1985). Transformation of *Salmonella* typhimurium with plasmid DNA: differences between rough and smooth strains. J Bacteriol.

[62] Clark AG, Glanowski S, Nielsen R, Thomas PD, Kejariwal A, Todd MA (2003). Inferring nonneutral evolution from human-chimp-mouse orthologous gene trios. Science..

[63] Nielsen R, Bustamante C, Clark AG, Glanowski S, Sackton TB, Hubisz MJ (2005). A scan for positively selected genes in the genomes of humans and chimpanzees. PLoS Biol.

[64] Chen SL, Hung CS, Xu J, Reigstad CS, Magrini V, Sabo A (2006). Identification of genes subject to positive selection in uropathogenic strains of *Escherichia coli*: a comparative genomics approach. Proc Natl Acad Sci U S A.

[65] Wachter J, Hill S (2016). Positive selection pressure drives variation on the surface-exposed variable proteins of the pathogenic *Neisseria*. PLoS One.

[66] Petersen L, Bollback JP, Dimmic M, Hubisz M, Nielsen R (2007). Genes under positive selection in *Escherichia coli*. Genome Res.

[67] Jonson AB, Normark S, Rhen M (2005). Fimbriae, pili, flagella and bacterial virulence. Contrib Microbiol.

[68] Liu CC, Ou SC, Tan DH, Hsieh MK, Shien JH, Chang PC (2016). The fimbrial protein is a virulence factor and potential vaccine antigen of *Avibacterium paragallinarum*. Avian Dis.

[69] Kim KH, Aulakh S, Paetzel M (2012). The bacterial outer membrane β-barrel assembly machinery. Protein Sci.

[70] Yeo HJ, Yuan Q, Beck MR, Baron C, Waksman G (2003). Structural and functional characterization of the VirB5 protein from the type IV secretion system encoded by the conjugative plasmid pKM101. Proc Natl Acad Sci U S A.

[71] Fercher C, Probst I, Kohler V, Goessweiner-Mohr N, Arends K, Grohmann E (2016). VirB8-like protein TraH is crucial for DNA transfer in *Enterococcus faecalis*. Sci Rep.

[72] Baron C (2006). VirB8: a conserved type IV secretion system assembly factor and drug target. Biochem Cell Biol.

[73] Davidson AL, Dassa E, Orelle C, Chen J (2008). Structure, function, and evolution of bacterial ATP-binding cassette systems. Microbiol Mol Biol Rev.

[74] Collatz E, Gutmann L, Williamson R, Acar JF (1984). Development of resistance to β-lactam antibiotics with special reference to third-generation cephalosporins. J Antimicrob Chemother.

[75] Galdiero F, Tufano MA, Galdiero M, Masiello S, Di Rosa M (1990). Inflammatory effects of *Salmonella* typhimurium porins. Infect Immun.

[76] Quezada CM, Hicks SW, Galán JE, Stebbins CE (2009). A family of *Salmonella* virulence factors functions as a distinct class of autoregulated E3 ubiquitin ligases. Proc Natl Acad Sci U S A.

[77] Isberg RR, Voorhis DL, Falkow S (1987). Identification of invasin: a protein that allows enteric bacteria to penetrate cultured mammalian cells. Cell..

[78] Koskiniemi S, Lamoureux JG, Nikolakakis KC, de Roodenbeke CTK, Kaplan MD, Low DA (2013). Rhs proteins from diverse bacteria mediate intercellular competition. Proc Natl Acad Sci U S A.

[79] Cao Z, Klebba PE (2002). Mechanisms of colicin binding and transport through outer membrane porins. Biochimie..

[80] Kovac J, Cummings KJ, Rodriguez-Rivera LD, Carroll LM, Thachil A, Wiedmann M (2017). Temporal genomic phylogeny reconstruction indicates a geospatial transmission path of *Salmonella* Cerro in the United States and a clade-specific loss of hydrogen sulfide production. Front Microbiol.

[81] Liu M, Douthwaite S (2002). Methylation at nucleotide G745 or G748 in 23S rRNA distinguishes gram-negative from gram-positive bacteria. Mol Microbiol.

[82] Gustafsson C, Persson BC (1998). Identification of the *rrmA* gene encoding the 23S rRNA m1G745 methyltransferase in *Escherichia coli* and characterization of an m1G745-deficient mutant. J Bacteriol.

[83] Achtman M (2004). Population structure of pathogenic bacteria revisited. Int J Med Microbiol.

[84] Smith NH, Hewinson RG, Kremer K, Brosch R, Gordon SV (2009). Myths and misconceptions: the origin and evolution of *Mycobacterium tuberculosis*. Nat Rev Microbiol.

[85] Sghaier H, Ghedira K, Benkahla A, Barkallah I (2008). Basal DNA repair machinery is subject to positive selection in ionizing-radiation-resistant bacteria. BMC Genomics.

[86] Hooper DC, Jacoby GA (2015). Mechanisms of drug resistance: quinolone resistance. Ann N Y Acad Sci.

[87] Farhat MR, Shapiro BJ, Kieser KJ, Sultana R, Jacobson KR, Victor TC, Warren RM, Streicher EM, Calver A, Sloutsky A, Kaur D (2013). Genomic analysis identifies targets of convergent positive selection in drug-resistant *Mycobacterium tuberculosis*. Nat Genet.

[88] Guibourdenche M, Roggentin P, Mikoleit M, Fields PI, Bockemühl J, Grimont PA (2010). Supplement 2003–2007 (no. 47) to the white-Kauffmann-Le minor scheme. Res Microbiol.

[89] Iqbal Z, Caccamo M, Turner I, Flicek P, McVean G (2012). *De novo* assembly and genotyping of variants using colored de Bruijn graphs. Nat Genet.

[90] Danecek P, Auton A, Abecasis G, Albers CA, Banks E, DePristo MA (2011). The variant call format and VCFtools. Bioinformatics..

[91] Croucher NJ, Page AJ, Connor TR, Delaney AJ, Keane JA, Bentley SD (2015). Rapid phylogenetic analysis of large samples of recombinant bacterial whole genome sequences using Gubbins. Nucleic Acids Res.

[92] Drummond AJ, Suchard MA, Xie D, Rambaut A (2012). Bayesian phylogenetics with BEAUti and the BEAST 1.7. Mol Biol Evol.

[93] Kumar S, Stecher G, Tamura K (2016). MEGA7: molecular evolutionary genetics analysis version 7.0 for bigger datasets. Mol Biol Evol.

[94] Tavaré S (1986). Some probabilistic and statistical problems in the analysis of DNA sequences. Lectures Math Life Sci.

[95] Kimura M (1980). A simple method for estimating evolutionary rates of base substitutions through comparative studies of nucleotide sequences. J Mol Evol.

[96] Baele G, Li WL, Drummond AJ, Suchard MA, Lemey P (2013). Accurate model selection of relaxed molecular clocks in bayesian phylogenetics. Mol Biol Evol.

[97] Baele G, Lemey P, Bedford T, Rambaut A, Suchard MA, Alekseyenko AV (2012). Improving the accuracy of demographic and molecular clock model comparison while accommodating phylogenetic uncertainty. Mol Biol Evol.

[98] Camacho C, Coulouris G, Avagyan V, Ma N, Papadopoulos J, Bealer K (2009). BLAST+: architecture and applications. BMC Bioinformatics.

[99] Gupta SK, Padmanabhan BR, Diene SM, Lopez-Rojas R, Kempf M, Landraud L (2014). ARG-ANNOT, a new bioinformatic tool to discover antibiotic resistance genes in bacterial genomes. Antimicrob Agents Chemother.

[100] Hyatt D, Chen GL, LoCascio PF, Land ML, Larimer FW, Hauser LJ (2010). Prodigal: prokaryotic gene recognition and translation initiation site identification. BMC Bioinform.

[101] Zhou Y, Liang Y, Lynch KH, Dennis JJ, Wishart DS (2011). PHAST: a fast phage search tool. Nucleic Acids Res.

[102] Li W, Godzik A (2006). Cd-hit: a fast program for clustering and comparing large sets of protein or nucleotide sequences. Bioinformatics..

[103] Tatusova T, DiCuccio M, Badretdin A, Chetvernin V, Nawrocki EP, Zaslavsky L (2016). NCBI prokaryotic genome annotation pipeline. Nucleic Acids Res.

[104] Li L, Stoeckert CJ, Roos DS (2003). OrthoMCL: identification of ortholog groups for eukaryotic genomes. Genome Res.

[105] Edgar RC (2004). MUSCLE: multiple sequence alignment with high accuracy and high throughput. Nucleic Acids Res.

[106] Suyama M, Torrents D, Bork P (2006). PAL2NAL: robust conversion of protein sequence alignments into the corresponding codon alignments. Nucleic Acids Res.

[107] Stamatakis A (2014). RAxML version 8: a tool for phylogenetic analysis and post-analysis of large phylogenies. Bioinformatics..

[108] RStudio Team (2015). RStudio: Integrated development for R.

[109] Conesa A, Götz S, Garcia-Gomez JM, Terol J, Talon M, Robles M (2005). Blast2GO: a universal tool for annotation, visualization and analysis in functional genomics research. Bioinformatics..

[110] Huson DH, Bryant D (2006). Application of phylogenetic networks in evolutionary studies. Mol Biol Evol.

[111] Martin DP, Murrell B, Golden M, Khoosal A, Muhire B (2015). RDP4: detection and analysis of recombination patterns in virus genomes. Virus Evo.

[112] Alvarez-Pérez S, de Vega C, Herrera CM (2013). Multilocus sequence analysis of nectar pseudomonads reveals high genetic diversity and contrasting recombination patterns. PLoS One.

[113] Liao J, Wiedmann M, Kovac J (2017). Genetic stability and evolution of the *sigB* allele, used for *Listeria sensu stricto* subtyping and phylogenetic inference. Appl Environ Microbiol.

[114] Yang Z (2007). PAML 4: a program package for phylogenetic analysis by maximum likelihood. Mol Biol Evol.

[115] Matute DR, Quesada-Ocampo LM, Rauscher JT, McEwen JG (2008). Evidence for positive selection in putative virulence factors within the *Paracoccidioides brasiliensis* species complex. PLoS Negl Trop Dis.

[116] Nielsen R, Yang Z (1998). Likelihood models for detecting positively selected amino acid sites and applications to the HIV-1 envelope gene. Genetics..

[117] Méric G, Yahara K, Mageiros L, Pascoe B, Maiden MC, Jolley KA (2014). A reference pan-genome approach to comparative bacterial genomics: identification of novel epidemiological markers in pathogenic *Campylobacter*. PLoS One.

[118] Benjamini Y, Hochberg Y (1995). Controlling the false discovery rate: a practical and powerful approach to multiple testing. J R Stat Soc Ser B.

[119] Chen H, Cohen P, Chen S (2010). How big is a big odds ratio? Interpreting the magnitudes of odds ratios in epidemiological studies. Commun Stat Simul Comput.

[120] Kruskal WH, Wallis WA (1952). Use of ranks in one-criterion variance analysis. J Am Stat Assoc.

