# Supplementary material for: Serotype-specific evolutionary patterns of antimicrobial-resistant Salmonella enterica
Source: BMC Evol Biol. 2019 Jun 21;19:132. doi: 10.1186/s12862-019-1457-5 (PMC6588947; doi:10.1186/s12862-019-1457-5)
Supplement: Supplementary file 2 — Figure S1. The distribution of AMR gene numbers of AMR S. Dublin, AMR S. Newport, and AMR S. Typhimurium isolates. The AMR gene number of AMR S. Dublin is significantly different from that of AMR S. Newport and AMR S. Typhimurium. Figure S2. The distribution of PPG gene numbers of AMR S. Dublin, AMR S. Newport, and AMR S. Typhimurium isolates. Figure S3. The distribution of pseudogene numbers in S. Dublin, S. Newport, and S. Typhimurium. Figure S4. Gene tree of AAC (6′)-Iaa inferred by maximum likelihood method. Tree is rooted by midpoint. Bootstrap values > 70% are presented on the tree. S. Dublin is indicated by blue, S. Newport by blue, and S. Typhimurium by red. Figure S5. Maximum likelihood tree of AMR S. Newport isolates, and Lineage II (sub-lineages - IIA, IIB and IIC) and Lineage III reference isolates. Tree is rooted by midpoint. Bootstrap values of major clades are presented on the tree. Reference isolates are indicated by red. (DOCX 681 kb) [file 12862_2019_1457_MOESM2_ESM.docx]

**Figure S1** The distribution of AMR gene numbers of AMR *S.* Dublin, AMR *S.* Newport, and AMR *S.* Typhimurium isolates. The AMR gene number of AMR *S.* Dublin is significantly different from that of AMR *S.* Newport and AMR *S.* Typhimurium.

**Figure S2** The distribution of PPG gene numbers of AMR *S.* Dublin, AMR *S.* Newport, and AMR *S.* Typhimurium isolates

**
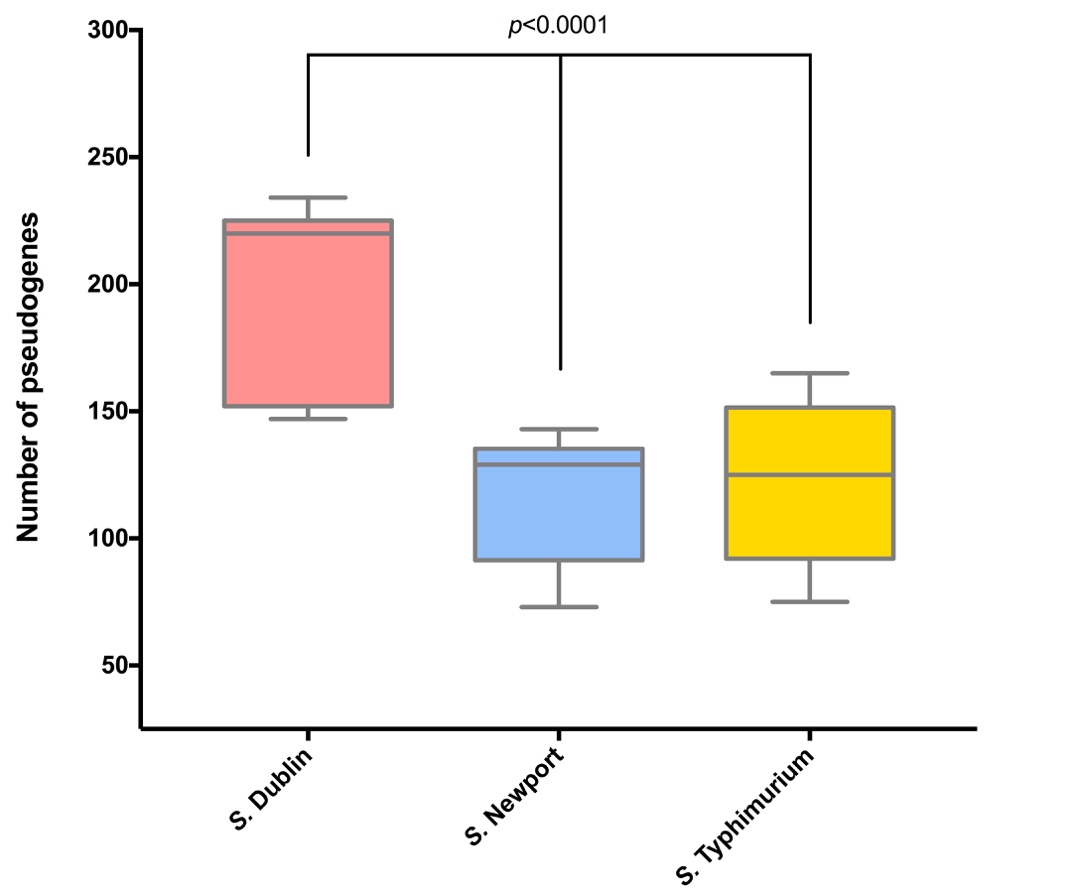
**

**Figure S3** The distribution of pseudogene numbers in *S.* Dublin, *S.* Newport, and *S.* Typhimurium.


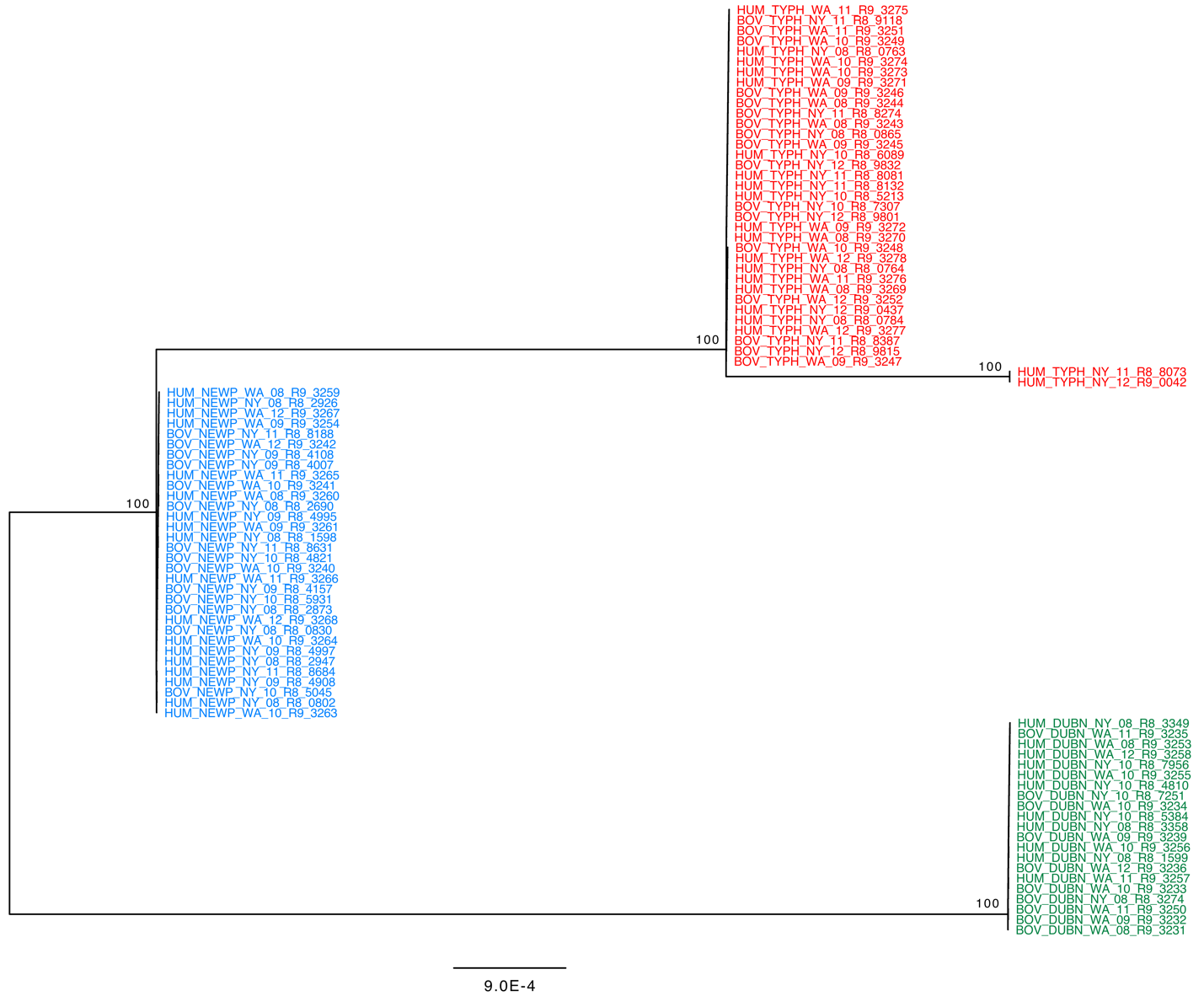


**Figure S4** Gene tree of AAC(6')-Iaa inferred by maximum likelihood method. Tree is rooted by midpoint. Bootstrap values >70% are presented on the tree. *S.* Dublin is indicated by blue, *S.* Newport by blue, and *S.* Typhimurium by red.

**Figure S5** Maximum likelihood tree of AMR *S.* Newport isolates, and Lineage II (sub-lineages - IIA, IIB and IIC) and Lineage III reference isolates. Tree is rooted by midpoint. Bootstrap values of major clades are presented on the tree. Reference isolates are indicated by red.
